# Towards Using Police Officers’ Business Smartphones for Contactless Fingerprint Acquisition and Enabling Fingerprint Comparison against Contact-Based Datasets

**DOI:** 10.3390/s21072248

**Published:** 2021-03-24

**Authors:** Christof Kauba, Dominik Söllinger, Simon Kirchgasser, Axel Weissenfeld, Gustavo Fernández Domínguez, Bernhard Strobl, Andreas Uhl

**Affiliations:** 1 The Multimedia Signal Processing and Security Lab, University of Salzburg, 5020 Salzburg, Austria; dsoellinger@cs.sbg.ac.at (D.S.); skirch@cs.sbg.ac.at (S.K.); uhl@cs.sbg.ac.at (A.U.); 2 Center for Digital Safety & Security, AIT Austrian Institute of Technology, 2444 Seibersdorf, Austria; Gustavo.Fernandez@ait.ac.at (G.F.D.); Bernhard.Strobl@ait.ac.at (B.S.)

**Keywords:** mobile biometrics, fingerprint recognition, fingertip segmentation, real-time application, performance evaluation

## Abstract

Recent developments enable biometric recognition systems to be available as mobile solutions or to be even integrated into modern smartphone devices. Thus, smartphone devices can be used as mobile fingerprint image acquisition devices, and it has become feasible to process fingerprints on these devices, which helps police authorities carry out identity verification. In this paper, we provide a comprehensive and in-depth engineering study on the different stages of the fingerprint recognition toolchain. The insights gained throughout this study serve as guidance for future work towards developing a contactless mobile fingerprint solution based on the iPhone 11, working without any additional hardware. The targeted solution will be capable of acquiring 4 fingers at once (except the thumb) in a contactless manner, automatically segmenting the fingertips, pre-processing them (including a specific enhancement), and thus enabling fingerprint comparison against contact-based datasets. For fingertip detection and segmentation, various traditional handcrafted feature-based approaches as well as deep-learning-based ones are investigated. Furthermore, a run-time analysis and first results on the biometric recognition performance are included.

## 1. Introduction

**Figure 1:**
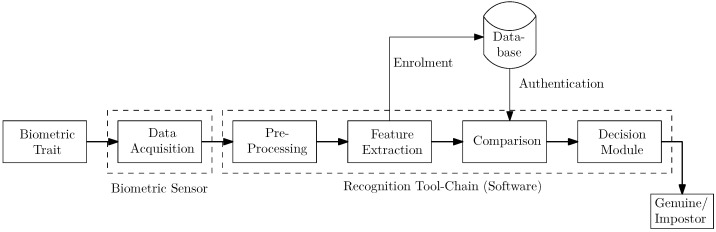
Biometric recognition toolchain.

Authorities responsible for the maintenance of public order, law enforcement, and civil security in the street (mainly police authorities) sometimes have to determine people’s identities. To establish the true identity of a person, technological advances offer authorities a wide range of possibilities. Most current techniques are based on biometric recognition technology. According to the ISO/IEC 2382-37 standard (https://www.iso.org/standard/66693.html, accessed on 1 March 2021), biometric recognition is the “automated recognition of individuals based on their biological and behavioural characteristics”. The most commonly used biological characteristics or biometric traits include faces, fingerprints, and irises. A typical biometric recognition system consists of several modules, as shown in Figure 1, where the first and most crucial step is the capturing of biometric sample data. Existing solutions require additional hardware to capture the data (e.g., capture devices in a vehicle and digital cameras for facial imaging). Afterwards, the captured data are processed and transmitted to the comparison system and a response will be sent back, and the whole process is expected to run in a reasonable time. Currently, there are only very few dedicated mobile fingerprint (FP)-capturing systems available to Austrian police officers. As a consequence, in order to establish the identity of a person, the police officer must transport the person to the nearest police station, where the FPs can be taken either using a contact-based optical scanner or using the classical ink-based technique. All in all, it can take up to one hour until the police officer is able to establish the person’s identity. Hence, establishing the identity of a person is a time-consuming task, and obviously, police forces and authorities wish to improve (speed up) this process.

Recently, biometric recognition systems have become available as mobile solutions or have even been integrated into modern smartphones. Most modern smartphones support FPs or face biometrics as a personal-identification method to unlock the device or to securely employ online-banking and e-commerce applications. This has led to the development of new and fast authentication/identification methods. In fact, smartphones are able to acquire and process FPs, enabling the use of the smartphone as an integrated biometric device. While most applications still depend on an integrated touch-based sensors, contactless acquisition of FPs utilizing the smartphone’s built-in camera has become feasible as well. Contactless acquisition exhibits several advantages, e.g., high user acceptance and the possibility to capture several fingers at once, but poses additional challenges, e.g., a more complex capturing process and a lower recognition performance in general.

The deployment of mobile devices (especially smartphones) might help authorities acquire the data for identity verification. Moreover, police officers often carry a business smartphone. Thus, by utilizing this device, biometric data can be acquired, processed, and transmitted so there is no need to carry an extra device. Although research on mobile biometrics has been carried out in the past, the recording quality, speed, and usability required for identification for civil authorities have not yet been achieved. Moreover, biometric-based security systems still have room for improvement (e.g., in their accuracy, their usability using mobile devices, and their scalability) due to the need for higher security and efficient usage. This need for higher quality and increased speed as well as usability was one of the main reasons why the project BioCapture (https://www.kiras.at/en/financed-proposals/detail/d/biocapture-biometrics-capture-tool-for-police-in-mobile-use/, accessed on 1 March 2021) was initiated. The project investigates the feasibility of a prototypical implementation for face and FP recording via a smartphone’s camera and its integration into the police workflow: acquisition of data, automatic quality analysis, format conversion, transfer to the recognition service’s headquarters, and triggering of a search for identity identification and a response that is sent back to the police officer. The final outcome of the project will be a mobile FP recognition solution with the following features:

- No additional hardware is needed to acquire the FP sample data. The phone’s built-in camera is used to capture the finger images.
- Four fingers are captured at once. Most existing solutions capture only one finger at a time. Our solution acquires one hand at a time (except the thumb) and automatically detects and segments the 4 individual fingertips prior to further processing. This saves a considerable amount of time during the acquisition process.
- After detection and segmentation of the fingertips, a FP enhancement is performed. This enhancement was specifically designed for contactlessly acquired FP images. As a consequence, a valid comparison with existing contact-based FPs (stored in a separate database) is enabled.
- The solution can be integrated into the current police workflow, in particular into the police AFIS (“Automated FP Identification System”). By defining suitable interfaces and format conversion, our approach can be integrated seamlessly into the current police workflow and used as an additional way of establishing the identity of a person rather than having to change the whole system to work with the mobile solution.
- The identification process, from data acquisition to presenting the result to the police officer, is designed to be user friendly. The police officer is guided through all the necessary steps, and clear instructions for what to do next are given on the phone.

Several commercial solutions covering most of the abovementioned features are available (details on these solutions are given in Section 1.1). The intended employment in the BioCapture project imposes several requirements that cannot be met by commercial solutions (details on these requirements are given at the end of Section 1.1). Hence, instead of employing an existing commercial solution, we resorted to developing a custom solution.

The focus of this paper is not on proposing a final solution that outperforms the existing competitors in terms of recognition performance. The main contribution of this paper is an engineering study covering an in-depth analysis and comprehensive evaluation of the main stages of the FP processing toolchain in the context of the BioCapture Project: data acquisition, FP preprocessing, and comparison of contactless FPs against contact-based ones. The FP preprocessing includes FP detection, segmentation, and image enhancement. For fingertip detection and segmentation, several traditional handcrafted feature-based algorithms as well as convolutional neural network (CNN)-based ones were tested. During the subsequent image enhancement, not only is a filtering-based enhancement performed but also a DPI (dots per inch) correction is applied first. Several different options are presented and evaluated for each stage. Based on the evaluation results, a recommendation for which option to employ is derived for each stage. These recommendations provide guidance towards the solution that is ultimately implemented and for further work on this topic. To the best of our knowledge, such a detailed in-depth study of all the different parts of the recognition toolchain has not been performed before.

Moreover, two datasets including FP images acquired in a contactless manner in real-life scenarios were used to evaluate the fingertip detection and segmentation approaches as well as the image enhancement approaches. The evaluation was done by comparing the enhanced contactless FP data against contact-based data from the same subjects using a commercial FP matcher. This evaluation shows that successful FP recognition is possible, which is essential because the final solution will be integrated into the police identification workflow. Furthermore, we investigated the feasibility of implementing these parts of a FP recognition toolchain on a smartphone: a prototype application to capture and process FP images in real-life scenarios was implemented and tested on a smartphone as the target platform. Currently, Austrian police officers are equipped with an Apple iPhone as their business smartphone. Thus, the iPhone 11 was chosen as the target platform. The application includes the FP capturing process (the police officer is guided through the capturing process to achieve good image quality), fingertip detection, fingertip segmentation, and FP image enhancement. We performed a computational cost analysis to highlight the differences compared to a traditional evaluation setup performed on a personal computer, including a GPU capable of performing CNN-based segmentation, and we benchmarked the accuracy of the FP segmentation provided by the different approaches, which are evaluated according to our smartphone-based segmentation protocol. We present the preliminary recognition performance results achieved with our contactless FP samples after segmentation and image enhancement and compare them with the results for contact-based samples, using state-of-the-art FP recognition systems, including an analysis of DPI-related issues.

The rest of the paper is organized as follows: Section 1.1 summarizes the related work and highlights the differences from existing systems as well as the necessary requirements of our targeted solution. Section 2 gives a description of the evaluation methodology, the FP segmentation algorithms used, and their implementation on the smartphone device as well as the datasets. The experimental setup and the results are presented in Section 3. A discussion of the results for each stage including recommendations on which option to employ in the final solution is given in Section 4. Section 5 gives our concluding remarks and outlines our next steps and planned improvements.

### 1.1. Related Work

During the last decade, many solutions for contactless FP recognition have been developed [1], which exhibit several advantages over contact-based ones, and there is an increased user acceptance, mainly due to reasons of hygiene but also due to the more unconstrained capturing environment, which is the most important advantage. Different processing chains are needed for smartphone cameras, including FP detection and segmentation as well as dedicated preprocessing methods. A survey of touchless FP technologies can be found in [2]. Furthermore, the same authors extended their survey in [3] by analyzing existing methods applied in the recognition process performed on selfie FP images. Apart from comprehensively discussing various techniques related to parts of the recognition workflow, they also compared the performance of state-of-the-art methods proposed in the literature. These state-of-the-art methods also include recognition systems based on recent deep-learning-methods, which were also part of a survey study by Minaee et al. [4]. The authors not only focused on FP biometrics but also included other biometric modalities (including ear, face, gait, iris, palmprint, signature, and voice), and they discussed several available datasets as well as deep-learning-methods regarding the strengths and main challenges of each modality. Lee et al. [5] have shown that face verification and FP recording are possible to some extent on smartphones. However, the accuracy largely depends on the image enhancement and quality restrictions of the underlying acquisition process. Stein et al. [6] presented a prototype for authentication of people on smartphones using finger-photo recognition, which captures FP samples using the built-in camera on the mobile phone at any orientation angle with respect to the camera. Operations for preprocessing are chosen with respect to both their computational effort and the accuracy achieved. The authors reported an Equal Error Rate (EER) of less than 20% if optimized parameters were used. With varying finger distance and less restrictions on image quality, considerable degradations are observed. Under laboratory conditions and using fixed finger distances from the smartphone, the EER can be improved to less than 5% [7,8]. Mac et al. [9] proposed a methodology for contactless FP recognition including segmentation and feature extraction as well as feature and data fusion and reported an EER of approximately 5%. Sankaran et al. [10] used a deep learning (DL)-based approach, a scattering network algorithm (ScatNet), for feature representation and comparison. They evaluated different existing FP recognition algorithms, focusing on photometric effects and performing experiments using various illumination conditions as well as two backgrounds (indoor and outdoor). EERs between 3.65% and 10.43% were reported.

All of the aforementioned contactless FP solutions aim to compare contactless FPs with contactless FPs, e.g., an authentication system implemented on a smartphone where the templates are generated from the contactless FP images and where, during authentication, a new template is generated based on a contactless FP image and compared with the stored one. We aim to provide a mobile FP-capturing tool for use by the police in the field, integrated into the current police workflow and AFIS system, which stores FP templates from flat-rolled contact-based FP samples. Hence, in order to be able to integrate our proposed solution, we need to be able to compare contactless FPs with contact-based ones.

#### 1.1.1. Contactless to Contact-Based Interoperability

The comparison of FP images captured by contactless sensors with FPs acquired by contact-based devices requires specific methods. This area of research is referred to as sensor interoperability, and it has turned out to be challenging but important. Specialized algorithms for contact-based [11,12,13,14] as well as contactless [15] sensors have been proposed. In the following, we focus on contactless to contact-based interoperability.

In a recent work by Libert et al. [16], an investigation regarding the interoperability of contactless to contact-based FP capturing systems was conducted. The authors established an in-house dataset containing contactless as well as contact-based FP samples from 200 subjects, acquired using several biometric capturing devices. Their evaluation using two different FP matchers showed that contact-to-contact performance is still best, followed by stationary contactless devices, while mobile contactless solutions performed less capably. In particular, control of scale (i.e. DPI) remains a challenge for mobile contactless solutions. Furthermore, they confirmed the hypothesis that multiple finger matching significantly improves the performance of contactless devices. Ericson and Shine [17] evaluated the interoperability and performance of contactless FP sensors compared with contact-based ones, using samples acquired during two different time periods. Depending on the sensor deployed, in some cases, they demonstrated true match rates of over 90%. Zhou et al. [18] evaluated the performances for a comparison of 3D FPs and the compatibility of 3D and 2D FPs. They reported EER values of between 0.00% and 1.38% in the case of a 3D–3D comparison, between 2.56% and 11.63% in the case of a 2D–3D comparison, and between 0.00% and 0.07% in the case of a 2D–2D comparison. Wild et al. [19] presented a FP segmentation algorithm based on the correlation of pixel RGB triplets and the evaluation of brightness and contrast, which can be applied to FP imprints captured with smartphones. Subsequently, they evaluated their segmentation method by comparing contactlessly acquired FP samples with contact-based ones, which resulted in an EER of 1%.

The following studies involve a dedicated contactless to contact-based mapping in order to improve the interoperability. Lin and Kumar [20] developed a contactless to contact-based approach to FP image mapping based on a generalized deformation correction model using a robust thin-plate spline transform. They achieved further improvements by incorporating minutiae-related ridges into the comparison process, which resulted in an EER as low as 4.46% on one publicly available dataset and 19.81% on a second one. The authors concluded that the current cross-matching error rates are not yet low enough for deployment. However, they reported that perspective distortion correction in contactless FP images is expected to reduce the error rates. In a direct follow-up paper [21], the authors proposed a DL-based approach to accurately match contactless and contact-based FP images. Deep FP representations were generated by a multi-Siamese CNN, which was trained on FP minutiae, their ridge maps, and specific regions of ridge maps. Their new approach outperformed previous approaches, resulting in EERs of 7.93% and 7.11% on the two datasets. In another follow-up work, Vyas and Kumar [22] developed a new approach to enhance contactless FP samples based on complementary ridge-valley information. They evaluated the cross-comparison performance using three standard minutiae-based matchers on the same two public databases as used in [20] and achieved EERs of 0.81% (original images) and 0.29% (inverted images) for the first database and 3.60% (original images) and 2.23% (inverted images) for the second one. Dabouei et al. [23] pointed out that the classical ridge enhancement methods fail to reconstruct the whole ridge map for contactless FP imaging. They proposed a deep model to rectify the perspective distortion of contactless FPs by combining a rectification and a ridge enhancement network. Their experiments on two public datasets resulted in an increase in the number of detectable minutiae. They were able to improve the EER on the second dataset used by [20] (19.81%) to 7.71%. Tan and Kumar [24] presented a more precise deep-neural-based minutiae extraction and a pose-compensation approach in order to address unwanted pose changes with contactless FP samples. Their proposed methodology is a three-step pose compensation framework, including view angle estimation, ellipsoid model formulation, and intersection area estimation. They evaluated their approach on the same datasets as used in [20,21] and reported EERs of 1.05% and 14.3% on the first and second datasets, respectively.

#### 1.1.2. Fingertip Detection and FP Segmentation

Detection and segmentation of the fingertip area is an important first step in FP recognition, and it is especially challenging for contactless FPs due to the complex and nonuniform background, light shading effects, and finger misplacement in all three dimensions.

FP segmentation for the latent FPs faces its own challenges and lessons learnt that can be transferred to contactless FPs. For the latent FPs, there are approaches based on ridge information (orientation field) [25], on the particular ridge structure (from coarse to fine) [26], on frequency information in the Fourier domain [27,28], as well as on a combination of ridge orientation and frequency information [29] and incorporating minutiae information [30].

For mobile or contactless fingertip detection and FP segmentation, which is of particular interest for the work presented in this paper, there are some simple approaches based on skin color information and segmentation from a controlled, uniform background [5,7,31,32,33]. A more sophisticated method is based on the mean shift algorithm and frequency/Wavelet transform information [34]. Labati et al. [15] aimed to achieve similar recognition accuracies to those obtained in touch-based systems in the case of a fully touchless one by employing a four-step finger segmentation procedure. They also addressed the interoperability of touchless with touch-based technologies and achieved an EER as low as 0.22% under laboratory conditions.

#### 1.1.3. FP Image Enhancement

The next crucial step in the processing toolchain is a proper FP-specific image enhancement. FPs are widely used in daily life; therefore, most aspects of successful enhancement have been already discussed in the literature for contact-based FPs [35]. A review of preprocessing methods for FP samples acquired in a touchless manner (captured by mobile phones) can be found in [36].

One important aspect that is not discussed in these studies is the influence of resolution changes. Knowing the correct resolution in terms of DPI for FP images is a crucial aspect when it comes to successful comparison not only with other contactless samples but also more importantly with contact-based ones. For images with an unknown DPI, several approaches exist to estimate this measure of image resolution. Most of them are based on FP ridge distance estimation, usually by applying a Fourier spectrum analysis approach, e.g., [37]. Kovacs-Vajna et al. [38] combined a spectral analysis scheme with a geometric one to calculate the average ridge distance and derived the DPI from the ridge distance. Sevakumar et al. [39] proposed a scheme to enhance the interoperability of several FP sensor types by performing an image comparison based on ridge distance averaging. Their experimental evaluation confirmed that the recognition performance can be improved if their proposed ridge-averaging scheme is performed after the thinning process and before the minutiae extraction. Yin et al. [40] evaluated a spectral analysis and statistical ridge distance estimation method as well as a combination of both methods (hybrid approach) regarding their capability in terms of robustness, accuracy, and computation time. They found that the hybrid approach was superior compared to any of the single ones.

#### 1.1.4. FP Recognition (Feature Extraction and Comparison)

The next steps in the processing toolchain include feature extraction and feature comparison in order to arrive at the final output (match/non-match). The most commonly employed systems are based on minutiae-related information [35]. As in many other fields, machine learning and DL methods have become increasingly popular and successful in the area of FP recognition [41,42,43,44]. In general, DL-based methods have either been applied to preprocess the raw FP samples [45] or to extract minutiae information [46,47,48]. More recent applications have focused on the preprocessing or minutiae extraction part of the biometric FP recognition toolchain, and some systems that are capable of performing the entire recognition process using raw FP samples have been proposed [43,49,50,51,52]. The most critical part of a fully automatized CNN-based FP recognition system is the minutiae extraction. In [52], an architecture was proposed, which segments FPs from their surrounding background and classifies the segmented FP samples into five different classes: arch, left loop, right loop, whorl, and tented arch. In [53], a patch-based Siamese CNN, which does not explicitly rely on the extraction of minutiae points, was designed and trained from scratch. The results obtained subsequently showed that the minutiae positions contained in the patches could be learned even without any explicit domain knowledge. However, as one of our requirements is to integrate our solution into the existing police workflow (AFIS system), we can neither change the feature extraction and comparison methods nor employ any of the CNN-based recognition approaches. Thus, they are not discussed further.

#### 1.1.5. Commercial FP Recognition Systems

Besides the academic approaches, there are several commercial companies that provide different types of FP recognition solutions. As we are interested in mobile and especially contactless solutions, we focus on these in the following. Several companies provide integrated contact-based FP sensors or add-on solutions for mobile platforms, e.g., FP cards AB (https://www.fingerprints.com, accessed on 1 March 2021), HID Global (https://www.hidglobal.com, accessed on 1 March 2021), Crucialtech (http://www.crucialtec.com, accessed on 1 March 2021), Idex (https://www.idexbiometrics.com, accessed on 1 March 2021), Integrated Biometrics (https://www.integratedbiometrics.com, accessed on 1 March 2021), and Precise Biometrics (https://www.precisebiometrics.com, accessed on 1 March 2021).

More recently, commercial companies have started to provide mobile contactless FP recognition frameworks for smartphone platforms. Diamond Fortress Technologies (https://www.diamondfortress.com, accessed on 1 March 2021) offers a software library named ONYX, which uses the camera of the smartphone to capture FPs. Their solution is able to capture four fingers at once and provides single-finger templates. Their library can be integrated into existing applications including FP image acquisition, segmentation, liveness detection, image export, and FP comparison on the device. Veridium (https://www.veridiumid.com, accessed on 1 March 2021) also offers biometric authentication using smartphones as a target platform. Their product “4 Fingers TouchlessID” provides multi-factor authentication using FP images captured by the smartphone. The software captures four FPs simultaneously, which are used during the authentication process. The template obtained is normalized to 500 DPI, and it can be exported for comparison with legacy databases. On 1–1 FP verification, Veridium claims False Rejection Rates (FRRs) less than 1.0% and False Acceptance Rates (FARs) of 0.01%.

The main goal of the BioCapture project is to develop a functional prototype of a mobile FP capturing tool for police field use that can be integrated into the current police workflow and AFIS system. This AFIS system stores FP templates from flat-rolled contact-based FP samples. Hence, in order to integrate the solution that is developed into the current system, a comparison of contactless with contact-based FPs is an essential part, taking interoperability aspects into account. Note that, from all of the approaches discussed so far, only two commercial products provide such a system as a single solution. These commercial products have reached a mature state, i.e., they are able to deliver sufficient recognition performance and they can be integrated into an existing system. Despite them being an adequate fit for our application scenario, there are some requirements imposed by the stakeholders of the BioCapture project prohibiting the deployment of those products. Hence, we had to resort to our own custom in-house solution rather than simply deploying an available commercial one. The reasons prohibiting the employment of an existing commercial solution and the advantages of in-house development are described in the following:

- One requirement imposed by the Austrian Ministry of the Interior is that the solution that is deployed must not add additional monthly costs per transaction or per client device. All eligible commercial products incur a monthly usage fee, at least per client, in addition to a considerable initial payment for the SDK and licence. For in-house development, there are only the initial development costs and the cost of ongoing maintenance efforts, and the latter is usually covered by in-house employees.
- Another important requirement imposed by GDPR and national laws is that the fingerprint images must not be transferred outside of the country (Austria). This not only includes the fingerprint sample images but also the features extracted from those images and even any clue that could potentially be linked to the subject’s identity. For commercial solutions, it is not clear what data are transferred to the manufacturer or stored for diagnosis reasons, prohibiting their application within the scope of the BioCapture project.
- In contrast to the commercial products, which are designed to work on different types/brands of smartphone devices, our in-house solution can be tailored to our single target platform, the iPhone 11, allowing for performance improvements (recognition performance, usability, and runtime) over the more generic commercial solutions.
- The source code of the commercial products is non-disclosed. Thus, future modifications and adaptations can only be made by the supplier, which usually involves additional costs and effort. For in-house development, the full source code is available, allowing for modifications and future adaptations/extensions.

The main contribution of this current paper is neither proposing a fully functional solution for mobile fingerprint recognition nor proposing a new approach to mobile fingerprint recognition that is able to outperform all of the existing ones. The character of the work presented here is an engineering study of mobile fingerprint recognition, including its problems and solutions to tackle these problems rather than proposing a final solution. The main contributions are the following:

- Providing a comprehensive and in-depth analysis of the different stages of the fingerprint recognition toolchain, including fingerprint sample image capturing, fingerprint preprocessing (segmentation and enhancement), and fingerprint adaptation (mapping/modification of contactless samples to make them compatible with contact-based ones).
- For each stage, several different methods are evaluated in terms of their respective performance, e.g., segmentation accuracy for the segmentation stage, and recognition performance for contactless to contact-based mapping.
- After the discussion of the results, a recommendation on which option to employ at each stage is given, which provides guidance for future work and serves as a basis for the final solution that will be developed during the BioCapture project.

Note that the two available commercial products meeting the requirements (except for the cost and data confidentiality requirements) are not included in our evaluations as, on the one hand, the single stages cannot be evaluated separately (these products are black-box solutions) and, on the other hand, they are not available to us (for “Onyx” from Diamondfortress, there is only a demo version with limited features available and, for “4 Fingers TouchlessID” from Veridium, there is no demo version at all).

## 2. Materials and Methods

**Figure 2:**
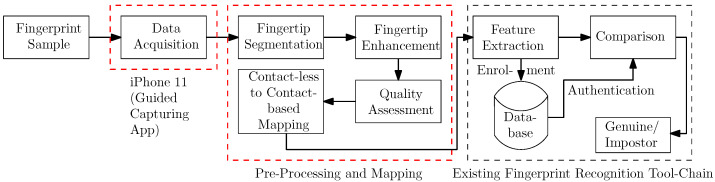
Contactless fingerprint (FP) prototype processing chain.

In this section, our contactless FP capturing and processing approach as well as the two datasets we acquired and used during the experimental evaluation are described. The processing chain can be seen in Figure 2 and consists of finger image acquisition, preprocessing and mapping, comparison, and the final verification/identification of the result. The preprocessing and mapping stage includes fingertip segmentation, quality assessment, fingertip enhancement, and contactless to contact-based mapping, which are the parts proposed in this paper, and they are surrounded by a red dashed line in the figure. Each of these modules is described in the following subsections, starting with image acquisition (using the default iPhone camera app as well as an acquisition app that we developed), followed by quality assessment, fingertip segmentation approaches (feature-based ones first, followed by the DL-based ones), FP image enhancement, contactless to contact-based mapping, and finally comparison and output of a decision.

### 2.1. FP Acquisition Software

**Figure 3:**
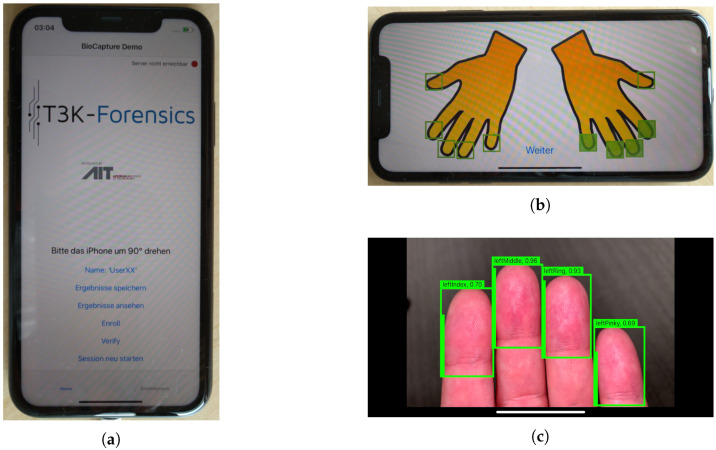
iPhone T3K app example images: (**a**) user interface, (**b**) selection of fingers before the acquisition starts, and (**c**) real-time detection and segmentation of the fingertips. Each fingertip is quantified by a simple quality measure, which is described in Section 2.4. If the FP quality is sufficient, all bounding boxes are colored green before the capturing process ends automatically.

The acquisition device used in the current study was an iPhone 11 (iOS 13, 8 Gb RAM, and 64 Gb storage capacity). In addition to the use of the default iPhone camera app, another acquisition solution (from now on named T3K app) was developed. The development of this custom app became mandatory because the standard iPhone camera app did not fulfill the requirements of a high-quality FP-capturing process, e.g., the auto-focus of the integrated application is not capable of accurately focusing on fingertips, especially if an inhomogeneous background is present, which is likely in outdoor data acquisition scenarios. The T3K app enables the focal length of the camera lens to be controlled (fixed), which simplifies the acquisition of high-quality FP images. Additionally, the camera light was set to be active during the entire capturing process with no regard to the ambient light conditions. This allows the influence of ambient light during the acquisition to be reduced, which is expected to enhance the quality of the samples captured. The T3K app features a graphical user interface as well as bundled software used for acquiring and processing the biometric data, which is captured as a video stream and can be output as videos or best shots per fingertip. Based on the video stream, FP detection and segmentation are performed in real-time. If the quality of the segmented FPs is high enough (above a pre-defined FP quality threshold), the app automatically ends the capturing process and stores the best shots (fingertip areas achieving the highest quality values according to the FP quality measure described in Section 2.4). Figure 3 shows some screenshots of the acquisition process.

Several end-user (i.e., police authority) and technical requirements needed to be addressed during design and implementation of the T3K app. The most important requirements are an intuitive user interface, ease of embedding into the existing police workflow (both end-user requirements), setting the focus of the iPhone camera to a fixed focal length, and allowing the fingertips to be captured as a video stream, which we discussed above (both technical requirements).

The intuitive user interface is necessary for two reasons. Firstly, a person whose identity needs to be verified presumably does not want to be identified and an uncooperative person will impede the FP capturing process, but the police officer must ensure a reasonably high capture quality such that the FP samples can be used in the identification process. In such a scenario, the officer will not have time to choose specific options potentially needed for the acquisition. Hence, only two steps are necessary before the acquisition can start. After starting the app, the officer must only turn the smartphone by $90^{\circ }$ and then select which fingers are to be captured (the default case is 4 fingers, missing the thumb). As soon as the selection is made, the capturing process starts automatically. Secondly, it is reasonable to assume that not all police officers will use the new app every day. Even after a training course, some of them are likely to forget how the app is used optimally. However if there are only the two steps mentioned above, through which someone using the app is guided each time, it is more likely that FP samples of reasonable quality will be captured, enabling a successful FP-based identification.

### 2.2. Fingertip Detection and Segmentation Approaches

Being able to detect different instances of fingertips in different lightning conditions quickly and robustly is a basic requirement for enabling the acquisition of high-quality FPs in real-world conditions. In general, fingertip detection can either be viewed as an object-detection or (instance-) segmentation problem. While object detectors are typically designed to produce a bounding box around an object (e.g., a fingertip), segmenters produce fine-grained masks, which provide a label for each pixel value. To address the problem of fingertip detection/segmentation, traditional handcrafted feature-based approaches—which is what we call the approaches not based on DL—as well as various DL-based approaches can be applied. In our case, the main challenge results from the fact that the chosen approach not only needs to be able to accurately detect fingertips but also has to reach real-time performance on a mobile device. Thus, checking the portability and tracing an approach’s performance is a crucial step before finally deciding on an approach.

In connection with the following description of the approach, it is important to note the assumptions being made regarding the acquisition scenario. As further FP comparison requires us to know the finger type (e.g., index finger) beforehand, we are required to infer not only the position of a fingertip but also its type from the detector/segmenter. As it turned out that detecting the finger type simply based on the characteristics of a finger (e.g., its shape) is almost impossible, we were required to constrain the way in which images are taken. As we did not want to force the user to manually provide the type of each finger, we instead decided to require valid images to always show exactly four fingertips. This way, together with the information on whether the left or right hand is presented to the smartphone, the finger type can be easily inferred for an arbitrary valid image. Images showing more or less than four fingers are considered to be invalid and are thus discarded during the image processing by the segmentation approach.

#### 2.2.1. Traditional Handcrafted Feature-Based Segmentation Approaches

In this section, three standard non DL-based approaches are described. All of them take a finger/hand image as input and output a segmentation of this image in the form of a binary mask, where pixels belonging to a finger/hand should be classified as fingertip pixels and all remaining parts of the image should be classified as background pixels.

##### Fast and Accurate Skin Segmentation in Color Images

The first traditional segmentation approach is based on the work of Tomaz et al. [54]. It is essentially a human skin segmentation approach, solely based on the color information of each pixel in the TSL chrominance color space. At first, an initial camera calibration is necessary to determine the color range considered as human skin color. Therefore, several sample images of different skin color and locations are taken and transformed in the TSL color space. The maximum and minimum S and T values of those images are determined and used to normalize/limit the S and T values of the images, which are to be segmented, leading to a normalized ST color space (the L component of the TSL color space is not used).

**Figure 4:**
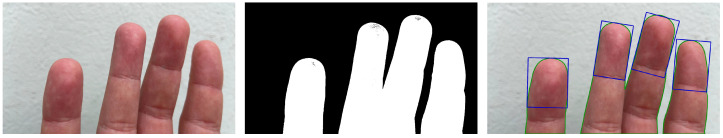
Example segmentation with [54] segmentation approach.

During the actual segmentation, prior to transforming the images to the TSL color space, a simple filtering based on color value is performed in the RGB color space to filter out areas with too much blue/green and those that are too dark, too yellow, or too green/blue in contrast to other colors. Afterwards, the filtered image is transformed to the TSL color space and then normalized to obtain a representation in the normalized ST color space. The actual segmentation is performed based on a simple thresholding using upper and lower bounds on the S and T values. These threshold values are derived from the observation that the scaled ST color space (the area of the ST color space to which human skin pixel values belong) can be represented by an ellipse in the normalized ST color space. After this thresholding, a median filter is applied to get rid of small, undesired isolated pixels that are not classified correctly. The last step is a grouping and connecting of similar regions based on their sizes and locations to close small holes in the segmented areas. The output is a binary mask, with the areas representing human skin as the foreground. An example image can be seen in Figure 4.

##### Color Histogram-Based Hand Segmentation

**Figure 5:**
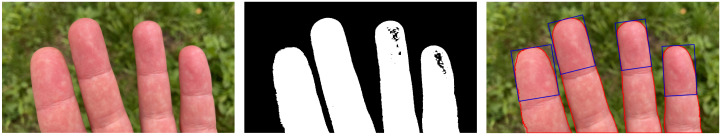
Example segmentation with a color histogram-based approach.

The second traditional approach again uses skin color information in order to segment the finger/hand from the background, together with some thresholding and filtering techniques to improve the segmentation results. The first step manually selects a reference area (rectangle), which has to be located inside the finger/hand area of the image. Afterwards, the image is transformed into the HSV color space. Then, a histogram is created from all pixels within the reference area (in the HSV color space), which is normalized using MinMax normalization. This histogram now contains information about the colors of the finger/hand within the given image and can be used to segment them from the background. This task is accomplished with the calcBackProject() function [55] provided by OpenCV, which works in a similar way to calculating a histogram, but instead of incrementing the respective histogram bin values, it reads the bin value and stores it. From a statistical point of view, it computes the probability of each element value with respect to the probability distribution, as represented by the image histogram, i.e., in our case, the probability of each pixel belonging to the finger/hand area. By thresholding this back-projection result, we obtain a segmentation of the image. To improve the result, an additional averaging filter is applied before thresholding. The output is again a binary mask, an example of which is shown in Figure 5.

##### Gaussian Mixture Model Background Subtractor

**Figure 6:**
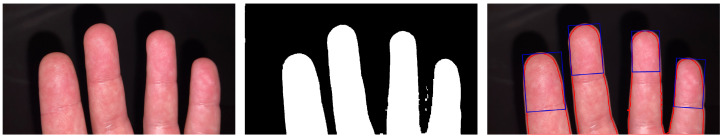
Example segmentation with the foreground/background subtraction approach.

The third traditional segmentation method uses one of the background subtraction methods provided by OpenCV [56], in particular BackgroundSubtractorMOG2 [57], which implements a Gaussian mixture-based foreground/background subtraction, as described in [58,59]. The only prerequisite is a background image of the scenery that has to be acquired separately. After that, the image that is to be segmented is presented, and based on the background image, a segmentation is performed, resulting in a binary foreground/background mask. Note that this type of background subtractor is intended for video/stream use. Hence, it has a learning algorithm and tries to update the background dynamically. As we only use it on a per-frame basis, we set the learning rate to 0 and do not dynamically update the background information. An example output image is depicted in Figure 6.

##### Finger Outline Detection and Bounding Box Fitting

All of the abovementioned traditional segmentation approaches output a mask with the finger/hand region as foreground and the remaining parts of the image as the background. Our aim is to position a bounding box around each fingertip and to then crop the fingertips based on the bounding box regions. Good locations of each min-area bounding box can be determined in the following way: The fingertip peaks can either be found by calculating the convexity defects of the convex hull around the segmented area or by finding the local extrema of the finger outline. As calculating the convex hull and the convexity defects is computationally expensive, we decided on the latter approach. Hence, after some mask enhancement (e.g., blob removal), we first identify the peak of each fingertip by transforming the mask into a series of y-values and by taking the maximum y-value belonging to a finger region in each row. Due to finger peaks being represented by local maxima in the series, we then use SciPy’s find_peaks() function [60] to find the series’ local maxima (and so the peak locations). Next, to fit a well-sized bounding box for each finger, the finger width needs to be estimated. Therefore, we first computes the finger contours using OpenCV’s findContours() method [61]. Knowing that the peak location needs to be a point in the contour list, we start from the peak point and collect all points along the contour until a certain height is reached (in our case, 0.15 of the image width). The finger width and orientation can now easily be determined by fitting a minimum-area rectangle using OpenCV’s minAreaRect() method [62]. The width of the rectangle obtained denotes the finger width. To obtain the final bounding box for fingertip cropping, we adjust the height of the rectangle (bounding box) to be 1.5 of the bounding box width. Having both the mask as well as the bounding box, we can finally obtain the segmented fingertip. These fitted bounding boxes are shown in the rightmost pictures in Figure 4, Figure 5 and Figure 6.

#### 2.2.2. CNN-Based Object Detection Approaches

In this section, the two CNN-based object detection models that directly output bounding boxes around individual fingertips are introduced, including details about the network structure and training of the models. The pretrained models are taken from the TensorFlow Object Detection API [63] and trained via transfer learning. We used the default hyperparameters as provided by the Object Detection API’s models and applied flipping, cropping, adjustment of brightness, as well as saturation and padding as augmentation techniques. For evaluation, both models were converted to a Tensorflow Light model.

##### SSD MobileNetV2

The Single Shot Multibox Detector (SSD) [64] is a rapid single-shot object detector for multiple classes, which uses MobileNetV2 [65] as its backbone. MobileNetV2 introduces two main features: linear bottlenecks between the layers and shortcut connections between the bottlenecks. The network is known to support real-time object detection with mobile devices. We use Non-MaximumSuppression (NMS) to ensure that not more than 4 objects are detected, since the maximum number of fingers per image is equal to four.

*Model choice and training*: The implementation used was based on the TensorFlow package’s Object Detection API [63], which offers a unified approach to multiple object detection frameworks that can run real-time inference on different platforms such as mobile phones. All training images were rescaled to 300×300 pixels and augmented, as proposed in [66]. The loss function used consists of two parts, the weighted smoothed L1 and the weighted sigmoid loss. While the former was applied for localization loss for bounding box offset prediction, the latter was applied for classification loss. The network was trained using the Root Mean Square Propagation (RMSprop) optimizer (*lr* = 0.004, decay = 0.9, momentum = 0.9, ϵ=1.0) with a batch size of 24 for 100 k iterations (≈2040 epochs). The validation dataset described in Section 2.8 was used to monitor the performance of the model during the training.

##### SSD MobileNetV2 Quantized

The SSD MobileNetV2 quantized model was based on the previously described SSD MobileNetV2 model (with NMS), but its weights were quantized. Quantization is the process of converting the model’s weights from floating-point numbers to low bit-width numbers such as 8-bit unsigned integers. This dramatically reduces both the memory requirement and computational cost of using the model.

*Model choice and training*: For the quantization, we retrained the model with a Quantization Aware Training (QAT) [67] from the TensorFlow Object Detection API. QAT simulates low-precision hardware during the neural network training process, adding the quantization error into the overall network loss metric, which causes the training process to minimize the effects of post-training quantization. We trained our model using RMSProp with a initial learning rate lr=0.004 (decay = 0.9, momentum = 0.9, ϵ=1.0) and a batch size of 24 for 200 k iterations (around 2040 epochs). Again, the validation dataset described in Section 2.8 was used to monitor the performance of the model during the training.

#### 2.2.3. CNN-Based Segmentation Approaches

In this section, the four CNN-based segmentation models used in this work are introduced. Networks with missing support for *instance* segmentation (SegNet and Deeplab) were trained to output a fine-grained mask of class labels from 0 to 4. The background is denoted by 0, the first fingertip in the image is denoted by 1, the second fingertip is denoted by 2, and so on. The area of each finger type can be easily found based on the class label. On the other hand, instance segmentation networks (Mask-RCNN) are able to distinguish between multiple instances of the same object, such as fingertips, out of the box. The type of a fingertip can then be inferred from the predicted object position.

##### SegNet

SegNet [68] is a fully convolutional deep encoder–decoder architecture for semantic pixel-wise segmentation. The encoder network is topologically identical to the 13 convolution layers in VGG16, and the decoder network is set up to mirror the encoder. To reverse the effect of the max-pooling operations in the encoder, max-pooling indices are stored and reused for upsampling in the decoder.

*Model choice and training*: The SegNet model used in this work was implemented by us but is topologically identical to the architecture proposed in the original work [68], which uses 3×3 convolutions, 2×2 max-pooling, and convolutional layers with 64, 128, 256, and 512 convolutional filters.

We trained our network on training images resized to 256×256 pixels. We refrained from using input augmentation since we could not see an increase in performance during various tests. For parameter optimization, the ADAM optimizer was applied (lr=0.001, $\beta_{1}\;=\;0.9$, $\beta_{2}\;=\;0.999$, $\epsilon \;=\;10^{-7}$) with a categorical cross-entropy loss. The network was trained for 80 epochs with a batch size of 15. After each training epoch, we measured its mean of intersection over union (mIoU) on the validation set (see Section 3.1.1) and kept track of the model performing best on the validation set. The model which performed best on the validation set was chosen as the final model and used to evaluate the performance on the test set.

##### Deeplab

Deeplab [69] is a network for semantic segmentation that relies on atrous convolutions to explicitly control the resolution at which feature responses are computed and effectively enlarge the field of view of filters to incorporate a larger context without increasing the number parameters. It uses so-called atrous spatial pyramid pooling (ASPP) to probe an incoming convolutional feature layer with filters at multiple sampling rates and effective fields-of-views, thus capturing objects as well as image context at multiple scales. In the latest version, DeeplabV3+ [70], a decoder module that refines the segmentation results along object boundaries was added.

*Model choice and training*: In this work, we relied on the official DeeplabV3+ implementation provided by Tensorflow (https://github.com/tensorflow/models/tree/master/research/deeplab, accessed on 1 March 2021). Due to the iPhone’s limited GPU memory, we decided to use MobileNet-V2 [65] as the feature extractor. A pretrained version of MobileNetV2 on ImageNet (which was also used in this work) can be found on the website.

We did not make any adjustment to the architecture itself, we only slightly adapted the training code to add support for our custom dataset. For this purpose, we changed the code to augment the training images with random scaling (scale factor: 0.5–1.5) and resizing to 513×513 pixels. For parameter optimization, we used an ADAM optimizer (lr=0.001, $\beta_{1}=0.9$, $\beta_{2}=0.999$, $\epsilon =10^{-8}$) and the cross-entropy loss. We finally trained the network for 500 epochs with a batch size of 20 and used the resulting model as our final model.

##### Mask R-CNN

Mask R-CNN [71] is a two-stage pipeline for *instance* segmentation. In the first stage, a convolutional neural network (CNN) backbone extracts features from the input image and class-agnostic region proposals are made. In the second stage, these proposals are refined and classified. For each proposal, segmentation masks are generated.

*Model choice and training*: In this work, we used a pretrained Mask R-CNN model with the ResNet-101 backbone and FPN [72] provided in the Detectron2 [73] framework. In both training and inference, we resized the input images to 1024×1024 pixels without changing their aspect ratios. During training, a multi-task loss [74] consisting of classification, a bounding box, and a mask loss for each sampled region of interest was determined. A synchronized SGD with an initial learning rate of 0.00025, a momentum of 0.9, and a weight decay of 0.0001 was used to train the model. The network was trained for 5000 iterations with a batch size of 2 (≈7 epochs). We used the default hyperparameters for training. No validation set was used during training.

##### HRNet

HRNet [75] is a network recently developed for human pose estimation and was later extended to support semantic segmentation [76,77]. While most semantic segmentation networks recover high-resolution representations from low-resolution representations, HRNet attempts to maintain a high resolution throughout the whole process. This was accomplished by combining multi-resolution (from high-resolution to low-resolution) parallel convolutions with repeated information exchange across parallel convolutions. High-resolution representations were then recovered from low-resolution representations using an upsample network (decoder) that is typically a mirror version of the encoder.

*Model choice and training*: The HRNet code used in this work is based on the official HRNet implementation (https://github.com/HRNet/HRNet-Semantic-Segmentation, accessed on 1 March 2021). Choosing HRNetV2-W18-Small as our reference architecture, the network has 4 stages. The first stage features a single block with 1 convolutional layer (32 filters), the second stage features 2 blocks with 2 convolutional layers (16 and 32 filters), the third stage features 3 block features with 2 convolutional layers (16, 32, and 64 filters), and the fourth stage features 4 blocks with 2 convolutional layers (16, 32, 64, and 128 filters). No pretrained model was used for training.

For training the network, we used multi-scale training with random scale factors between 0.5 and 2.1 and images sizes of 480×480 pixels. For parameter optimization, we used SGD with an initial learning rate of 0.01, a weight decay of 0.0005, and a momentum of 0.9. An exponential decay with a power of 0.9 was used to decay the learning rate over time. We trained the network for 400 epochs with a batch size of 20 using the cross entropy loss. In the same way as for SegNet, we kept track of the best-performing model by measuring the mIoU on the validation set in each epoch and we chose the best-performing model as the final model.

### 2.3. Portability and Runtime Analysis

The FP segmentation/detection approaches used were initially developed in Python under LINUX. However, since the main aspect of the BioCapture project is the development of a mobile app for contactless FP capturing and recognition, it is important to investigate the portability and the runtime of the approaches introduced above on the iPhone 11. Thus, the training models were converted into ML Core files and ported to the iPhone. The computer vision algorithms written in Python were converted into C++ code. The corresponding challenges and issues including the runtime analysis are discussed in detail in Section 4.3.

### 2.4. Finger Image-Quality Assessment

The FP image-quality assessment in our processing chain is responsible for ruling out all those segmented FP images that do not have sufficient quality before the enhancement is performed. The method used is based on a Canny edge detector. After applying the edge detector, the gradient of each pixel in the image is compared with a predefined threshold. The number of all pixels with a gradient above the threshold is summed up and set in relation to the image size. This results in a simple but efficient measure of sharpness for our application. If the sharpness is sufficient, the image is further preprocessed.

### 2.5. FP Preprocessing/Contactless FP Enhancement

**Figure 7:**
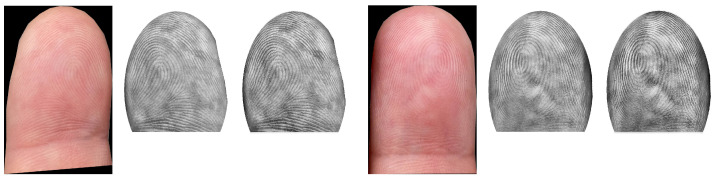
Example images of FP images after applying the enhancement approaches without inverting the grey values. After the enhancement, the ridges in the contactless FPs are shown as brighter structures.

**Figure 8:**
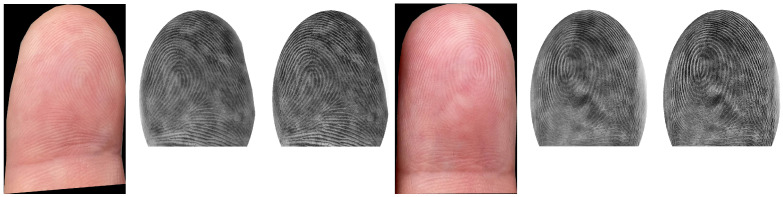
Example images of FP images after applying the enhancement approaches with inversion of the grey values. After the enhancement, the ridges in the contactless FPs are shown as darker structures. As a consequence, these preprocessed images look more similar to contact-based FPs than the non-inverted FPs (See Figure 7).

FP preprocessing is a crucial part of a FP recognition toolchain. In our given situation, we need to perform an enhancement of the contactlessly acquired FP ridge and valley structure in order to achieve the best possible compatibility with contact-based FPs. We investigated different approaches, but the visual results and the recognition performance were only promising for two of them (both closely related to each other). Example images are given in Figure 7 and Figure 8.

Before introducing the details of the enhancement approaches investigated, we want to discuss two features that both methods have in common: *area adaptation* and *grey value inversion*. Area adaptation is performed in all cases, but grey value inversion is optional. The area adaptation ensures that the contactlessly captured and enhanced FP images have similar shapes to the one they would have if they had been acquired in contact-based manner. Therefore, an elliptical mask is generated and applied to the FPs.

As we already know, it is likely that the contactless FP images were acquired under varying lighting conditions (see the dataset description for further details), which also affects the visibility of the ridge–valley structure. In the worst case, the ridge–valley structure can be inverted due to shadowing. The grey value inversion should compensate for that. In a practical scenario, the given light condition (outside: sun, inside: artificial light) will certainly not affect the entire fingertip during the capturing process but most likely some parts, depending on the position of the mobile phone camera. Hence, inverting the FP image can be a good option to enhance the recognition performance, but it is also possible that not inverting is the better solution. Additionally, a mixture of both strategies could be a valid solution. In this case, some regions of the FP would not be inverted and others would. However, it might be that the grey value inversion has a positive influence on only one enhancement method, and a dataset-dependent influence is also possible. Note that we plan to address this inversion issue in more detail in a future study. The details of each of the enhancement approaches are as follows:

(A) *bilateral filtering + contrast-limited adaptive histogram equalization (CLAHE):* The first approach is the simpler of the two enhancement strategies. First, an input FP image is converted to a greyscale one. Then, area adaptation is performed and optionally grey-value inversion can be applied, followed by horizontal flipping, which is necessary due to the differences in contactless and contact-based acquisition. The contactless samples resemble a horizontally mirrored version of the contact-based ones, which needs to be compensated for. As a second step, bilateral filtering is performed, which is highly effective at noise removal while preserving edges. The reason for selecting this filter is an obvious one: we want to preserve the separability between ridges and valleys by maintaining an unaltered edge structure and to simultaneously remove artefacts introduced during the capture, which tend to be problematic during the FP comparison. After the filtering, a contrast-limited adaptive histogram equalization (CLAHE) [78] is applied to enhance the contrast of the greyscale image.
(B) *average filtering + CLAHE + image sharpening:* The second approach starts with the same procedure as the other enhancement method-greyscale conversion, area adaptation, and horizontal flipping. The only difference for this procedure is if grey value inversion is applied. In that case, the area adaptation is performed as the very last part of the enhancement. If the area adaptation is not performed as the last part of the enhancement, the FPs are blurry because of the subsequent average filtering. Before applying CLAHE, an average filtering is also performed. The average-filtered image is used to de-blur the input FP by subtracting the filtered image from the greyscaled and flipped imprint. After applying CLAHE to enhance the contrast, an additional image sharpening using a common 3×3 kernel is performed.

### 2.6. Contactless to Contact-Based Mapping

Comparing contactless and contact-based FP images with sufficient accuracy cannot be done without any adaptations or a specific mapping. This mapping needs to compensate for different variations that are introduced during the contactless data acquisition, including longitudinal and transverse fingertip rotations, fluctuations of distance from the camera, and variations in resolution, which can lead to different values. Combined with the influence of different lighting conditions already mentioned, a challenging mapping task needs to be performed. In this paper, we do not focus on these issues. We want to answer the question of whether it is possible to perform a reasonable FP comparison without implementing a dedicated contactless to contact-based mapping. Such a mapping will be part of a future paper. Hence, one main objective of the investigation was to establish a baseline that does not include a specific contactless to contact-based FP adaptation. Thus, our investigation follows a similar protocol to the one used in the NIST report on interoperability assessment [16].

Nevertheless, the DPI variations are a major issue that needs to be considered, especially in the contact-based and contactless FP comparison. Typical minutiae-based frameworks are optimized for contact-based scanners, where the distance between the finger surface and the scanner surface is constant and known. Thus, all images captured with one and the same scanner exhibit the same resolution in terms of DPI. For contactless FP images, the distance between the finger surface and the image sensor varies; thus, a variety of different DPI can occur. The main problem in this case is that the minutiae points are extracted at different positions depending on the image resolution. As a consequence, the extracted minutiae points will not match even if images of the same subject and the same finger are compared because the comparison software does not expect different DPI resolutions to be present.

To compensate for varying DPI levels, several approaches can be used. In the work presented in this paper, only a preliminary interoperability analysis was carried out. Hence, a dedicated DPI estimation and compensation was not performed. Instead, we applied a simple comparison-score-based DPI compensation. From the acquisition settings, we knew the approximate distance between fingertips and camera as well as the anticipated DPI resulting from the size of the acquired images. Thus, this rough DPI approximation was used to select the most plausible DPI settings for each dataset.

### 2.7. FP Comparison

In our targeted use case (integration into the current Austrian police workflow), a comparison between contactless and contact-based FP samples is performed. To obtain a first impression of the possible recognition performance, we conducted a comparison experiment using a commercial state-of-the-art FP recognition system (Neurotechnology VeriFinger matcher *‘VeriFinger SDK 11.1’* (http://www.neurotechnology.com/verifinger.html, accessed on 1 March 2021)) to extract the FP templates and to compare them using the Fingerprint Verification Contest (FVC) protocol [79]. Ideally, each FP image should exhibit a resolution of 500 DPI, but in our case, we do not know the exact DPI due to acquisition variations. Using the newly developed T3K app, the DPI variation is limited to about 5% due to the fixed focal length. For the iPhone app, variations of up to 50% are possible. To compensate for the DPI variations, as described in Section 2.6, the following DPI levels were used: 750, 850, and 950 DPI in the case of T3K app data while 400, 450, and 500 DPI in the case of iPhone camera data. Three sets of comparison scores were obtained for each dataset and used as input for an EER-based performance evaluation: the lower the EER for a specific DPI selection, the more appropriate the selected DPI value is for the dataset. Those DPI levels were used as an input to the VeriFinger matcher, which performs an internal DPI adaptation based on the information provided.

In many applications, only index and middle fingers are used during comparison. Obviously, the more fingers that are included, the higher the accuracy. Especially if a more detailed analysis of the FPs is necessary, e.g., to perform a comparison against latent FPs acquired at a crime scene, the remaining fingers of a subject are also taken into account. We therefore also included a per finger/hand analysis in our performance evaluation.

### 2.8. AIT Dataset

In total, two different datasets were acquired in the course of the current investigation. Both were acquired under similar but slightly varying conditions, which gave us the opportunity to test the robustness of our segmentation and enhancement approaches.

The first dataset was acquired at the Austrian Institute of Technology (AIT) premises in Vienna during June 2020. A total of 15 subjects were recruited with balanced demographic characteristics in terms of gender and age: 66.67% of the subjects were men, and 33.33% were women; 13.33% of the subjects were between 20 and 30 years old, 26.67% were between 30 and 40, 33.33% were between 40 and 50, and the remaining 26.67% of the subjects were above 50 years of age. Most of the subjects were Caucasians (93.33%) and only one was dark-skinned.

**Figure 9:**
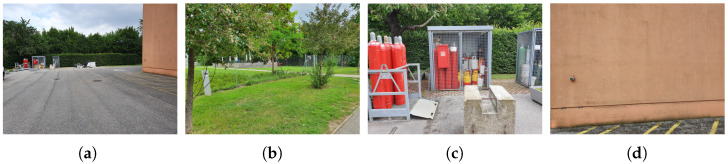
Some of the scenarios where data were recorded: (**a**) scenario number 3 (street), (**b**) scenario number 4 (grass), (**c**) scenario number 5 (lattice), and (**d**) scenario number 6 (wall).

**Figure A1:**
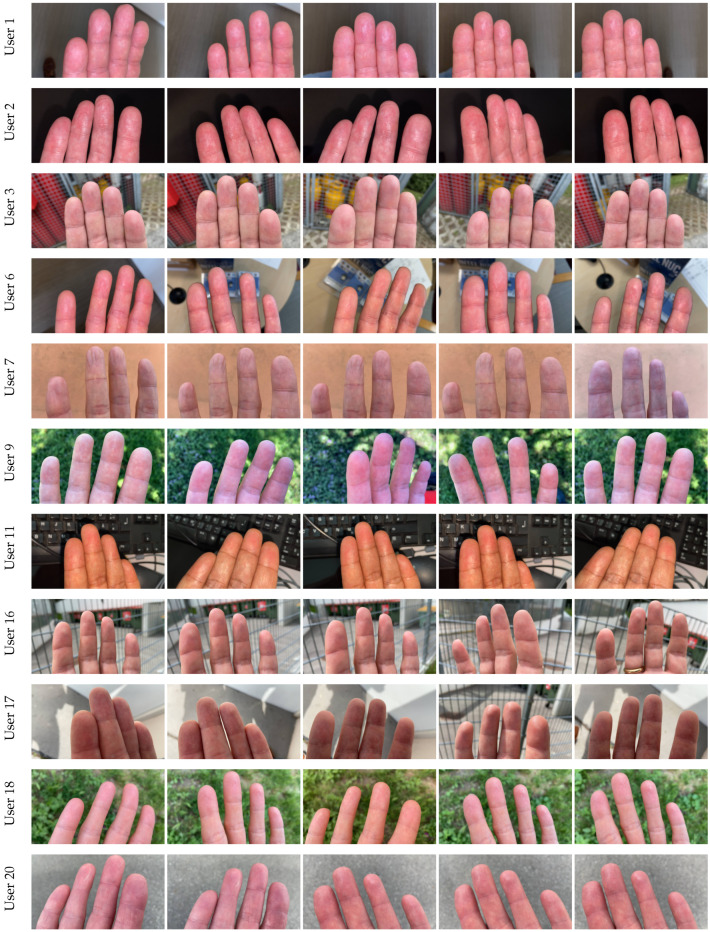
Sample frames from the Austrian Institute of Technology (AIT) dataset.

**Figure A2:**
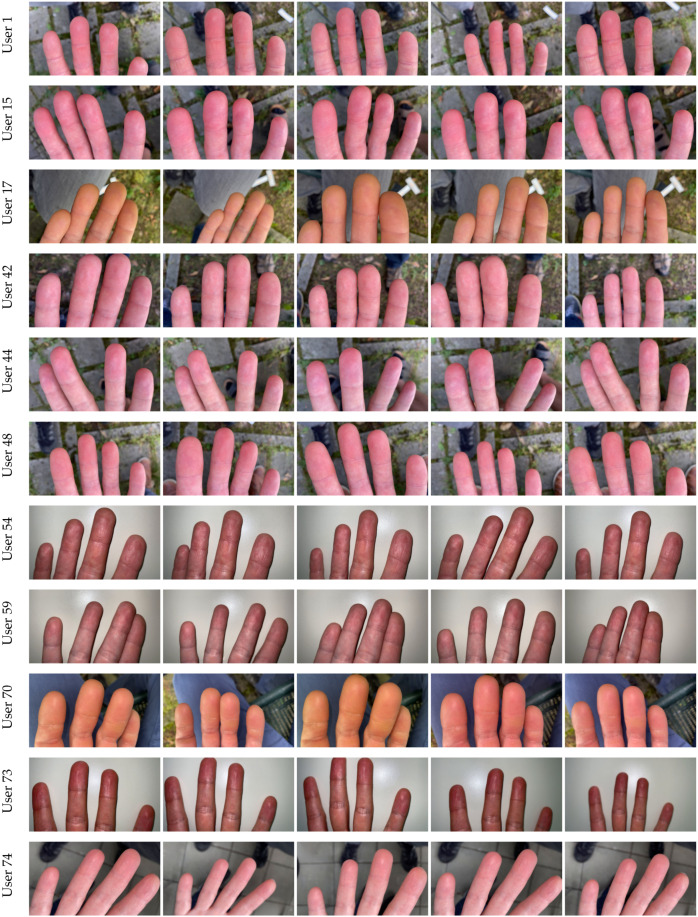
Sample frames from the PLUS dataset captured with the T3K app.

Seven scenarios were defined for acquiring the data: two scenarios were in an office-like environment, four scenarios were open-air, and the last one was in a cellar to simulate a night situation. Table 1 summarizes the main features of each scenario, and Figure 9 depicts some of them. Figure A1 shows some examples (one row per subject) of the acquired images to highlight the intra-class variations in the selected subjects. The corresponding segmented fingertips are illustrated in Figure A2.

**Figure 10:**
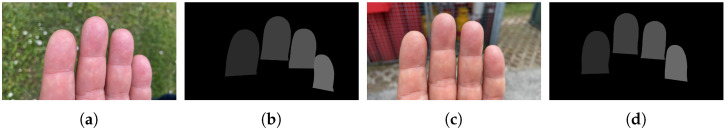
Finger samples: (**a**,**c**) original images, (**b**,**d**) mask images. (**a**,**b**) scenario number 4 (grass), and (**c**,**d**) scenario number 5 (lattice).

The acquisition was carried out using an iPhone 11 in combination with the T3K app, as described in Section 2.1. For each scenario and each participant, each hand was recorded on a separate video, which means a total of 210 videos were recorded (210=7×15×2). Each video had a length of between 10 and 15 s and a frame rate of 30 fps, and they were stored in .mov format and had a resolution of 3840 × 2160 pixels. A total of 2128 frames were extracted from all of the videos, equally distributed over all the scenarios, and the fingertips in each frame were manually annotated. The annotated frames were used to train and evaluate the fingertip detection and segmentation approaches presented in Section 2.2. The set of fingertip images used for interoperability analysis (Section 3.2.2) was obtained by selecting every fifth video frame and by extracting the individual fingertips using the Deeplab model described in Section 2.2.3. For each finger, the 10 highest-quality shots were selected, based on the quality assessment method described in Section 2.4. As a result, the AIT dataset is composed of 1200 contactless fingerprint sample images in total. However, since contact-based samples were only available for 11 subjects, only 880 contactless fingerprint images could be used for interoperability analysis. Examples of the acquired images and the corresponding annotation masks are shown in Figure 10. Details of the annotation protocol can be found in Section 3.

The FP scanner used to acquire contact-based samples was the DactyScan84C (hereafter named DS84c) produced by the Italian company GreenBit (https://www.greenbit.com/, accessed on 1 March 2021). The device is an FBI-certified appropriate. ten-print live scanner that acquires FP data in two modalities (four slaps and rolled fingers) and has acquisition speeds of 28 fps and 25 fps for slap fingers and rolled fingers, respectively. The acquired FP images (single slap imprints) have a resolution of 500 DPI and 256 grey levels. The acquisition process was carried out indoors under artificial lighting conditions (office room conditions, i.e., around 400 lux).

### 2.9. PLUS Dataset

The PLUS dataset is the second dataset used in the current study. It was acquired at the University of Salzburg during September 2020. A comparable amount of contactless FP data from 17 people (4 female and 13 male participants) was acquired under conditions compliant with privacy protection; 5.88% of the subjects were younger than 30 years old, 41.17% were between 30 and 40, and the remaining 52.94% of the subjects were between 40 and 50 years old. Two scenarios were defined for acquiring the data: one scenario was an office-like environment, and the other scenario was an outdoor scene. In the first one, there was artificial ambient light and the fingertips were captured in front of a homogeneous texture (desk surface) in the background, while in the second scenario, the ambient light conditions were a sunny day and the background was inhomogeneous and included grassy areas and stone tiles.

**Figure A3:**
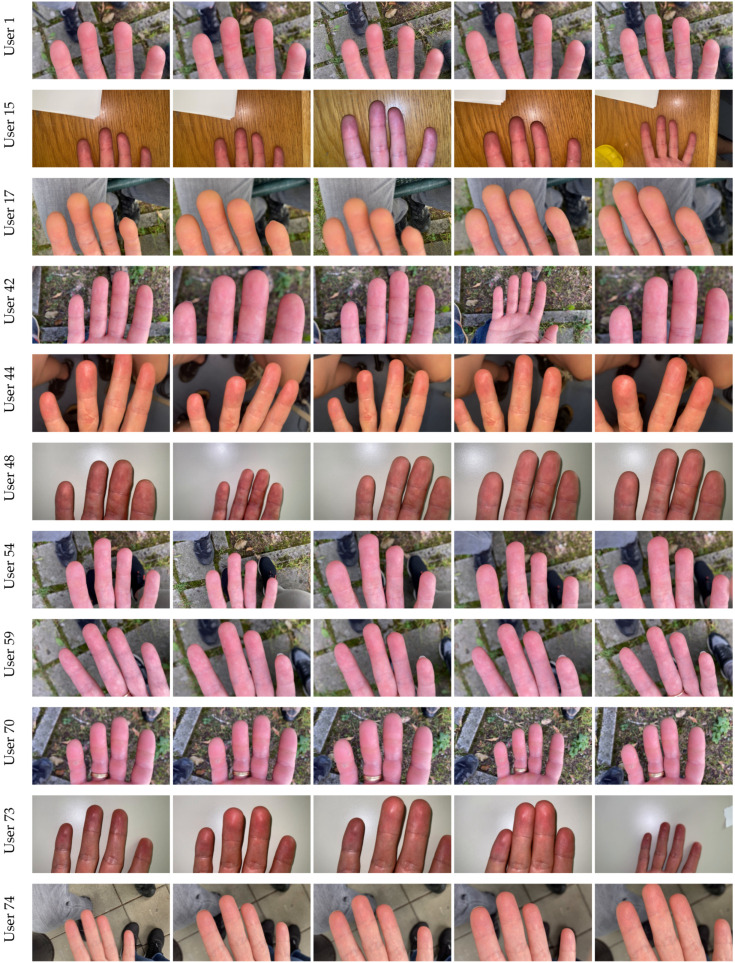
Sample frames from the PLUS dataset captured with the iPhone camera app.

Four subsets were included in the PLUS dataset, but only two of them were captured using the T3K app. Thus, these two subsets were subject to the same capturing restrictions as the AIT dataset and were therefore ruled out. The other two subsets were acquired using the standard iPhone 11 camera app, which leads to a less restricted capturing process and results in more challenging data as there is no fixed distance and focus for the app. As a consequence, there are more degrees of freedom for hand and finger placement (with respect to the camera). This allows for more longitudinal and tangential rotations as well as tilts, and the amount of perspective distortion is higher. Several of these variations are depicted in Figure A2 and Figure A3 for the T3K App and the iPhone camera app, respectively. For instance, in the third row (User 17) of Figure A2, an in-planar hand rotation as well as a tilt of the fingers towards the camera is present. In the fifth row (User 44), spreading of the fingers as well a varying tilt towards the camera causing perspective distortions is noticeable. In addition to the aforementioned variations, out-of-focus fingertips and varying distances between the fingers and the camera can been seen in Figure A3 (especially visible for Users 17, 48, and 74). These less-constrained acquisition conditions were allowed in order to test the robustness of the proposed segmentation and enhancement pipeline. We measured the robustness indirectly using a state-of-the-art FP recognition program by comparing the segmented and enhanced FP images with contact-based FPs of the same fingers captured by two optical FP scanners.

**Figure 11:**
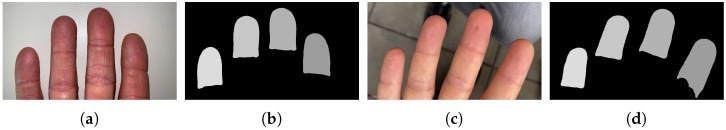
Finger samples: (**a**,**c**) original images, (**b**,**d**) corresponding mask images. (**a**,**b**) Indoor scenario, and (**c**,**d**) outdoor scenario.

**Figure A5:**
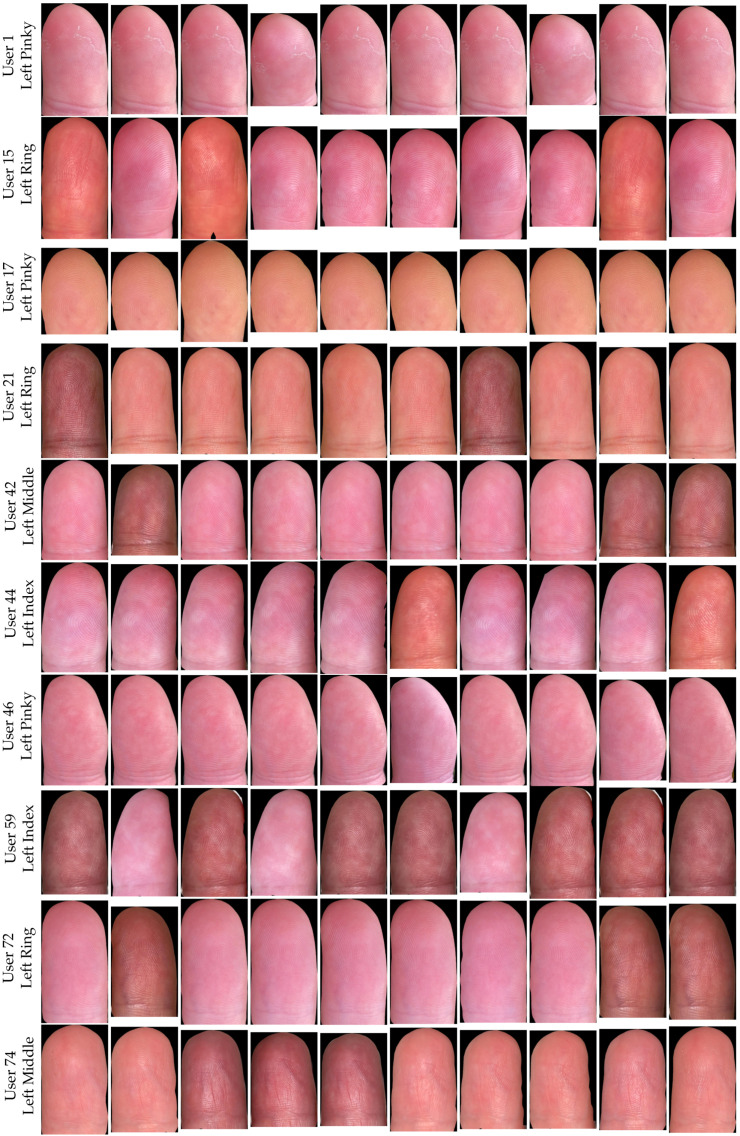
Fingertip samples obtained from the PLUS dataset captured using the T3K app.

**Figure A6:**
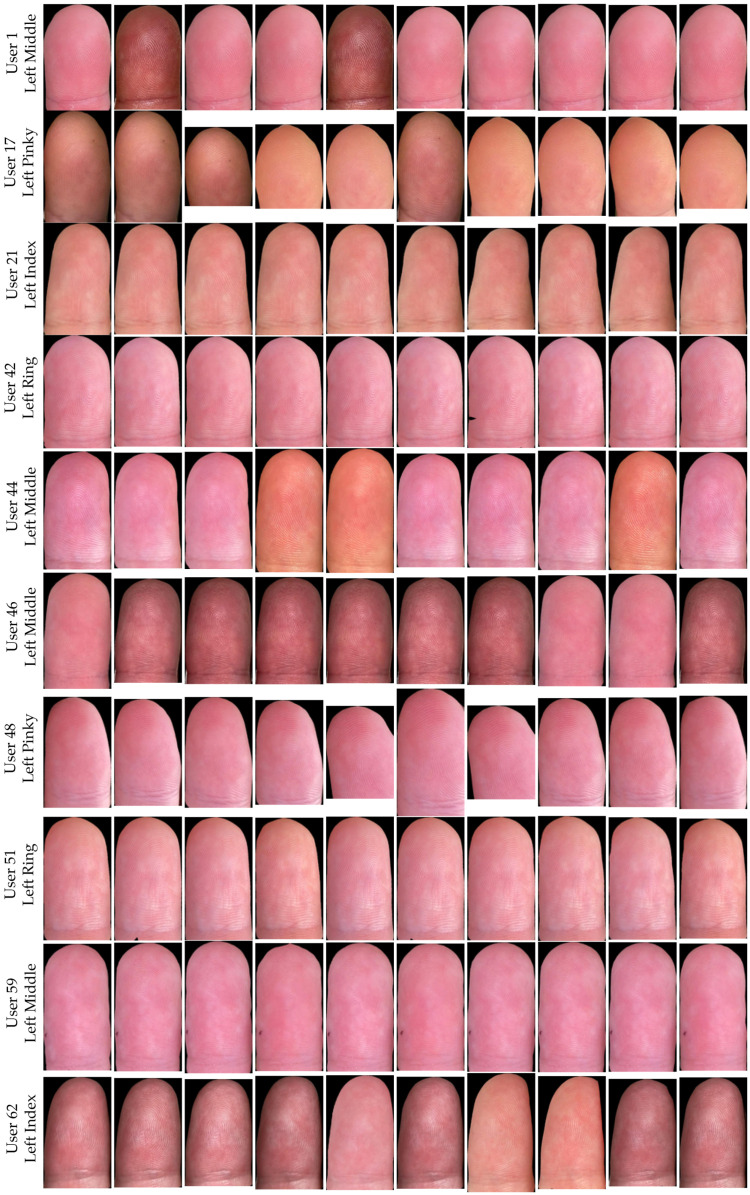
Fingertip samples obtained from the PLUS dataset captured using the iPhone camera app.

In total, the PLUS dataset consists of 136 videos (136=2scenarios × 17subjects × 2hands ×2acquisition apps). Each video has a length of about 40 s and a frame rate of 30 fps and is stored in .mov format. Videos captured with the T3K app have a resolution of 3840 × 2160 pixels. Videos captured with the standard camera app have 1920 × 1080 pixels. To obtain the set of fingertip images used for interoperability analysis (Section 3.2.2), every fifth video frame was selected and individual fingertips were extracted using the Deeplab model described in Section 2.2.3. For each finger, the 10 highest-quality shots were selected based on the quality assessment method described in Section 2.4. As a result, the PLUS dataset is composed of 2640 contactless fingertip images in total, 1320 fingertip images captured with each app. Some examples are shown in Figure 11. Some selected examples of the segmented fingertips, which were used to evaluate the recognition performance, are depicted in Figure A5 and Figure A6.

**Figure 12:**
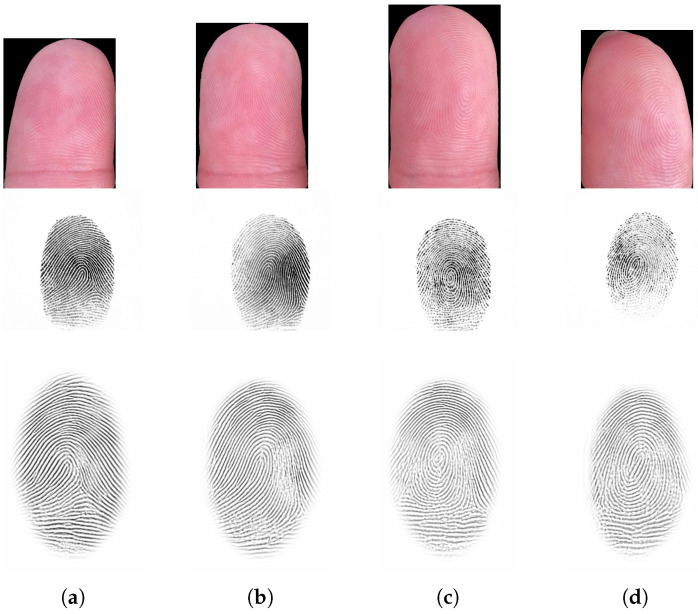
Examples of corresponding contactless and contact-based FP images. The top row shows contactless images acquired indoors, the middle row shows FPs acquired by the RealScan-G1 scanner, while the bottom row shows prints of the same fingers acquired by the Lumidigm V311. The leftmost image (**a**) is from the index finger, the next is from the middle finger (**b**), the one after that is from the ring finger (**c**), and the rightmost one belongs to the pinky finger (**d**).

The first scanner used during the contact-based FP image acquisition was the RealScan-G1 (denoted as G1 in the following) produced by the South Korean company Suprema (https://www.suprema-id.com/en/contents/detail.php?code=020105, accessed on 1 March 2021). This device was an FBI PIV certified single-finger live scanner, which is also IP54-rated, and it was contained in a dust and waterproof housing. The FP images acquired have a resolution of 500 DPI. The second scanner was a Lumidigm V-Series FP reader (https://www.hidglobal.com/products/readers/single-finger-readers/lumidigm-v-series-FP-readers, accessed on 1 March 2021), the Lumidigm V311 (denoted as V311 in the following), which was produced by the US company HID Global. It includes multispectral imaging technology that allows FP images to be captured when there are difficulties in distinguishing FP features (dirty, aged, dry, wet, moist, or incorrectly placed fingers). The resolution of the FPs that are acquired is once more 500 dpi. In total 680, FP images were captured with each of the devices deployed. The acquisition process was carried out indoors under artificial lighting conditions. Examples of corresponding contactless and contact-based FPs are shown in Figure 12.

## 3. Results

This section is divided into three subsections. The first one comprises a description of the CNN training protocol and the segmentation evaluation method. The second subsection contains the segmentation and recognition performance results. The last subsection comprises a runtime analysis of the segmentation approaches (performed on the iPhone 11).

### 3.1. Evaluation Protocol

In the following, the CNN training protocol and dataset composition are described, followed by an explanation of the segmentation performance metrics.

#### 3.1.1. CNN Training and Dataset Composition

Since CNN-based segmentation and object detection models always require data to be trained on, we first split the AIT dataset (see Section 2.8) into training, validation, and test sets. In order to keep the test set as realistic as possible and to avoid incorporating information from specific users in the training set, data were split according to their user ids. The first seven users from the dataset were used for training, the last seven users were used for testing, and the remaining users were used for validation. As a result, the training, validation, and test datasets consisted of 1176 images, 168 images, and 784 images, respectively.

All CNN-based segmentation approaches were then trained on the training set. Further details of the training of the different networks can be found in Section 2.2.2 and Section 2.2.3.

To generate training and test data for the approaches that yield bounding boxes rather than segmentation masks (e.g., R-CNN or the feature-based methods), we converted each mask into a set of bounding boxes by fitting a straight bounding box around each fingertip.

**Note:** As we found all fingertip detection/segmentation approaches to have difficulty distinguishing between left and right hands, we decided to simplify the fingertip detection task by removing the hand type information from the masks to not require the network to learn the type of hand. To accomplish this, we replaced the eight finger- and hand-specific class labels with four fingertip labels which were assigned based on the order of appearance of the finger in the image from left to right. Note that the hyperparameters reported were tuned empirically on the validation set.

#### 3.1.2. Object Detection and Segmentation Performance Metrics

We evaluated the object detection/segmentation performance of the different fingertip detection approaches using the *mIoU* (mean of intersection over union) metric [80]. The *mIoU* is one of the standard metrics used to assess the performance of segmentation models. As in our case some fingertip detection approaches yield bounding boxes rather than masks, the *mIoU* does not seem to be applicable for evaluating the performance of each of the proposed approaches at first glance. Metrics such as the *mAP* (mean average precision) seem to be better suited for this type of approach. Nevertheless, we still decided to rely on the *mIoU* for assessing the performance (even for object detection models) due to various reasons: Firstly, bounding boxes can be easily converted into a set of binary masks by plotting each bounding box on a zero mask and by setting pixels within the bounding box to one. Having a set of binary masks, the performance of the object detection approach can then be measured using the *mIoU*. Secondly, calculating the *mAP* requires a prediction’s confidence score, which is unavailable in the case of the feature-based segmentation approaches presented in Section 2.2.1. The same is the case for the segmentation approaches when converting the masks to bounding boxes. In this case, we lack a confidence score for the bounding box that is generated. Thus, we preferred the *mIoU* over metrics such as *mAP*, which does not require a confidence score.

Note that, for the segmentation approaches, the *mIoU* is simply calculated across all five available output maps (four finger masks + background masks). For the object detection (bounding box) approaches, the *mIoU* is calculated across all four predicted bounding boxes maps. If less or more than four bounding boxes are predicted, we treat the bounding box predictions as invalid as we are not able to uniquely assign a finger type (e.g., index or middle finger) to the bounding boxes. Bounding boxes are therefore are replaced by four zero maps to reflect the invalid prediction in the final *mIoU* score.

### 3.2. Experimental Evaluation

First, the fingertip detection/segmentation results are presented, followed by the interoperability recognition performance results. Afterwards, the results for the more detailed per finger/hand analysis are given, and finally, the runtimes of the different segmentation methods are given.

#### 3.2.1. Fingertip Detection/Segmentation Results

To compare the performance of the object detection and segmentation approaches presented in Section 2.2, the performance of each model is evaluated on the test set. The quality of the predicted masks and bounding boxes measured using the average *mIoU* is presented in Table 2. As can be seen in the table, segmentation approaches were trained and tested not only on RGB (column “RGB”) but also on greyscale (column “greyscale”) images. The *mIoUs* for the detection/segmentation approaches were calculated by training/testing the network five times. Note that, for the traditional handcrafted feature-based approaches, this was not possible, as they specifically rely on features exclusively available in the RGB domain. The results are discussed in Section 4.1.

#### 3.2.2. Interoperability (Recognition Performance) Results

At first, the contact-based datasets using all FP images were evaluated using the VeriFinger SDK to establish a baseline biometric recognition performance given in terms of EER in percent for subsequently obtained interoperability recognition results: DS84c ≈0.00%, G1 ≈0.00%, and V311 ≈0.23%. The DS84c and G1 datasets enabled perfect recognition, while the V311 dataset still achieved a very respectable recognition performance.

Table 3 presents the recognition results using all FP images for each scenario, grouped by the enhancement method applied. The recognition experiments are denoted by the contact-based device abbreviations. There are two columns per dataset, corresponding to whether grey value inversion was performed. The AIT dataset only contains samples captured using the T3K app; hence, there are no results for the iPhone app, and the corresponding entries are marked with a diagonal line. It can be seen that the best DPI setting for the AIT dataset is 850. For the PLUS dataset, the selection is influenced by the choice of the contact-based scanner and the chosen acquisition application: 400 DPI (iPhone app) or 950 DPI (T3K app) if G1 is used and 450 DPI (iPhone app) and 950 DPI (T3K app) if V311 is applied. The results are discussed in Section 4.5.

#### 3.2.3. Detailed Finger/Hand Evaluation Results

After determining the best DPI setting for each dataset and enhancement method, the per finger/hand evaluation was conducted with the best DPI settings selected. Only data acquired by the T3K app for both the AIT and the PLUS dataset were used during this evaluation (no iPhone app samples for the AIT dataset). The results are listed in Table 4, starting with the numbers for the left and right hands individually. As motivated in Section 2.7, both the index and middle fingers are often used; hence, their combination was evaluated separately. The numbers for the evaluation of each single finger are then given. The results are discussed in more detail in Section 4.5.

#### 3.2.4. Analysis of Computational Performance

To understand whether the approaches presented allow for real-time fingertip detection/segmentation on the iPhone 11, we measured the average time required by two approaches (Deeplab and SSD MobileNetV2 Non-Quantized) for annotating a single frame. Details of how these models were ported to the iPhone and what prevented us from porting the other approaches can be found in Section 4.2.

For the purpose of measuring each model’s computation time, we developed a tiny iPhone app that uses the same modules and models as the original application. Taking the recorded videos presented in Section 2.8 as input, the application measured the time to annotate each video frame. During the experiment, we took care to ensure that no other applications were running in the background. For each participant (15 people), 2 videos (left hand and right hand) of each scenario were analysed. The minimum, maximum, median, and mean values and the standard deviation were then calculated for each video. Table 5 shows the statistics obtained by averaging the computation times of the videos of each scenario (indoor, outdoor, and all). The results obtained are discussed in Section 4.3.

## 4. Discussion

In the following, the results for all modules of our proposed mobile contactless FP recognition system are discussed with respect to the practical feasibility of the overall system for use by the police force in the field.

### 4.1. Segmentation Results

As can be seen from Table 2, Mask R-CNN (0.956) and Deeplab (0.94) trained on RGB images are the winners of the segmentation approaches tested in this work. SegNet RGB (0.9) and HRNet RGB (0.92) also provide good segmentation results but perform slightly worse on average. Furthermore, it can be seen that networks trained on greyscale images rather than RGB images perform almost equally well. Only in the case of HRNet does the grayscale network perform considerably worse. In any case, we can recommend using RGB over greyscale images. Looking at the fingertip detection task (also known as the bounding box location), we can see that the best-performing approaches are SSD MobileNet Quantized Greyscale (0.915) and Mask R-CNN RGB (0.949). Taking into account that, in contrast to the other segmentation approaches, they are directly trained using bounding box information, seeing this behavior is not surpising.

Looking at the traditional handcrafted feature-based approaches, we can see that all of them performed considerably worse than the other approaches tested. Besides that, they turned out to be considerably slower in our experiments. After careful investigation into why they performed so poorly, we found that, in many cases, these approaches detected more, or less, than four fingertips on a single image. As explained in Section 3.1.2, in this case, no finger type can be assigned and all bounding box predictions have to be discarded. Note that, in the case of “Fast and accurate skin segmentation”, 60% of the predictions had to be discarded. In the case of the “Gaussian Mixture Model” and “Color histogram segmentation”, the figure was even larger. This fact considerably decreased the performance of all traditional handcrafted feature-based approaches. The better performance of traditional approaches in the mask case is again a direct consequence of more, or less, than four fingertips being found on an image. When calculating *mIoUs* in the mask case, discarded fingertip detections are counted as zero masks. For this reason, *mIoUs* increased considerably in the mask case.

Although both object detection approaches (SSD MobileNet and SSD MobileNet Quantized) lag behind the performance of Deeplab and Mask R-CNN, they performed well enough to be considered for practical use for fingertip detection. It is interesting to see that the quantized version performs equally well (if not better) than the non-quantized version. Hence, we can conclude that the increased memory consumption of the non-quantized version is not required for better fingertip detection performance.

### 4.2. Portability

As explained in Section 2.1, the acquisition software has to run on an iPhone 11. For this reason, it was not enough to only investigate the detection/segmentation accuracy of each approach. Portability and runtime aspects also had to be considered. We therefore attempted to port the six CNN-based detection/segmentation approaches to measure their runtime on the iPhone 11. Note that we decided against porting the traditional approaches due to their bad detection accuracy, making them inadequate for practical use. Although frameworks such Tensorflow claimed to have good support for the application on mobile devices, porting the various models turned out to be a tricky task. For instance, in the case of Segnet, Mask R-CNN, and SSD MobileNetV2 Quantized, we failed to convert the model to CoreML format due to model operations not supported by CoreML. While at least from a technical point of view, it should be possible to work around this problem by adding these missing operations as custom layers developed by us, we refrained from doing so because of the effort and complexity. Besides that, we expect Mask R-CNN to run fairly slow on the iPhone 11 due to its huge model size. Mask R-CNN has about 63.3 M parameters, while SSD MobileNet only has about 4.6 M parameters. A similar problem occurred when we tried to convert the HRNet model to CoreML format. Unfortunately, the root cause (perhaps of a bug in the coremltools toolbox) never became fully clear. Taking into account that we finally managed to get SSD MobileNet (object detection approach) as well as Deeplab (segmentation approach) up and running on the iPhone 11, we waived more detailed investigations. The conversion of the SSD MobileNet tensorflow model is based on a script (https://github.com/hollance/coreml-survival-guide/blob/master/MobileNetV2%2BSSDLite/ssdlite.py, accessed on 1 March 2021) provided by [81], which consists of three steps. In a first step, it converts the trained tensorflow model to Core ML by stripping away unused subgraphs as well as by performing built-in preprocessing operations, and the non-maximum suppression and the model’s weights are converted to 16-bit floats. A second subgraph converts the predictions to real coordinates, which can be processed by a non-maximum suppression model. Finally, these three models are combined into a single-pipeline model. The Deeplab implementation is based on the official model provided in the Tensorflow Model Garden (https://github.com/tensorflow/models/tree/master/research/deeplab, accessed on 1 March 2021). Converting the model to CoreML using coremltools (https://github.com/apple/coremltools, accessed on 1 March 2021) turned out to be straightforward. Note that, due to the poor performance of the traditional handcrafted feature-based approaches, we omitted the portability and runtime evaluation for these approaches, i.e., these approaches were not ported to the iPhone 11.

### 4.3. Runtime

Table 5 reveals that, regardless of the approach, the computation time is independent of the scenario (thus, the environment in which the frame is captured). From a technical point of view, this behavior is not surprising since computation times mainly depend on the operations performed during a model’s forward pass. Since the model remains the same in both scenarios (indoor/outdoor), computation times can also be expected to be similar.

Besides that, we are interested in the question of whether both approaches achieve real-time performance (∼25 fps, not less than 20 fps). In the case of Deeplab, we can see that the worst-case computation time is ∼45 ms ( ∼22 fps), the best-case computation time is ∼35 ms (∼29 fps), and the average computation time ∼39 ms (∼26 fps). In the case of SSD MobileNetV2, the worst-case computation time is ∼43 ms (∼23 fps), the best-case computation time is ∼21 ms (∼48 fps), and the average computation time is ∼25 ms (∼40 fps). Hence, both approaches satisfy the requirements for real-time fingertip annotation.

### 4.4. Trade-Off between Quality, Portability, and Runtime

Luckily, we found the two successfully ported approaches (Deeplab and SSD MobileNet) to be among the best approaches. Furthermore, it turned out that there is also no limitation from a runtime point of view. Both approaches are able to annotate images in real-time.

Therefore, the question that remains is which of the two approaches should be used in the final app. Taking into account that the fine-grained masks provided by Deeplab allows for perfect segmentation of each fingertip, Deeplab might at first seem to be the best choice. However, there is a trade-off that becomes evident once both approaches are tested in the final app. In a real-world scenario, SSD MobileNet appears to be much more robust than Deeplab. As Deeplab has to provide a class label for each pixel, there is always a chance of wrongly annotating some pixels. Therefore, some additional postprocesssing is required to detect and clean these pixels. We therefore decided to use SSD MobileNet for our final app and to use a dedicated foreground/background segmentation network operating in the backend, trained to segment the fingertips that are detected if necessary.

### 4.5. Contactless to Contact-Based Recognition Performance Results

The DPI settings of 850 for the AIT dataset, 400/950 DPI for the PLUS dataset G1, and 450/950 DPI for the PLUS dataset V311 were optimal in terms of recognition performance (see Table 3). The recognition performance is not only influenced by proper DPI selection but also by acquisition application and the corresponding datasets. At first, we assumed that the fingertip images from the iPhone’s standard photo app would result in a more challenging dataset (due to more degrees of freedom). However, this assumption could not be proven because variations introduced during the acquisition—and not by the capturing app choice—had a more severe impact. These conditions include variations according to the scenarios that were defined (including different environmental conditions) and the way in which the iPhone was moved in relation to the subject’s hands (different operators telling the subjects what to do). Overall (baseline and interoperability experiments as well as finger-specific results), for the PLUS dataset, the comparison against G1 data outperformed the V311.

In general, the performance for both PLUS datasets is inferior to the data captured by the T3K app. The best EERs for the PLUS standard camera app data are 5.87% and 4.21% using the T3K app, while an EER of 1.65% was obtained for the AIT dataset. Hence, the T3K app is better suited for practical application, mainly due to the limited range of possible DPI levels. In the current study, we have performed only a very rough DPI correction for all of the datasets, and thus, the DPI settings might not be absolutely precise. This inaccuracy affects the biometric recognition process and leads to a degradation (increase) of the obtained EER values. Note that the exact DPI level can vary from image to image, is not handled by our current approach, and has a negative impact on the recognition performance.

Furthermore, the selected enhancement strategy has a dataset-specific impact. For both contactless datasets, the second enhancement strategy (average filtering + CLAHE + image sharpening) is beneficial in most cases, especially for the best DPI setting. As a consequence, this enhancement scheme is the preferred choice. Although the bilateral filter is designed to preserve edge information (representing the ridges) while simultaneously reducing noise, simple image sharpening turned out to be more effective in our case. Grey value inversion, inverting ridges and valleys, had a beneficial impact on both the AIT dataset and the PLUS ones, but the overall performance increase was limited. In fact, for the best performing DPI settings, especially on the AIT dataset, the difference between inversion and no inversion is quite small, while for both PLUS data subsets, an improvement in recognition performance can be seen. As mentioned in Section 2.5, ridges/valleys may be inverted in some parts of an image while they are not inverted in others. During data acquisition for the current study, all subjects participated voluntarily and were fully cooperative. However, a subject examined by the police may not be cooperative, resulting in deliberate finger/hand movements and other issues during the acquisition. This would degrade the image quality and complicate the enhancement process, and it needs to be addressed in future work.

The results of the evaluation of the single finger/hand combination gave some additional insight into which FPs might cause the poor performance of contactless FPs compared with the contact-based baseline. It turned out that the selection of the hand has an impact in all experiments. The performance of the right hand is superior. One possible explanation is that most subjects participating in the study are right-handed. In addition, not all fingers of the right hand performed better than the corresponding ones from the left hand. In the case of the left index finger, it can be seen that the EER value is (a) better for the DS84c sensor and (b) worse for the PLUS datasets. On the PLUS dataset using the G1 sensor, the recognition performance of the right index and middle fingers outperformed all the others.

If each finger is considered independently, the left ring/pinky finger as well as the right index/pinky one performed worst compared with all others, and this is the main reason for the performance loss on the PLUS datasets. On the AIT dataset, the left middle and both pinky fingers are responsible for the observed performance degradation compared to the baseline results. In general, the ring and especially the pinky finger lead to reduced performance for contact-based samples [35]. In particular, during the acquisition of our data sample, the index and middle fingers were located in the centre of the image, which was on the focal plane. Consequently, the chance that the ring/pinky finger was out of focus was higher compared with the index and middle fingers, leading to reduced performance. Nonetheless, in some cases (e.g., right index finger in the G1 dataset) an almost perfect recognition accuracy (0.00% EER) was achieved on the PLUS dataset, and on the AIT dataset, five of the eight fingers that were separately investigated resulted in an EER lower than 0.25%. This is a very promising starting point for further investigation.

### 4.6. Recommendations towards a Final, Fully Integrated Solution

In the following part of the results discussion, we summarize the most important findings for each stage of the recognition toolchain and give recommendations for future work towards the final working prototype:

- *FP Image Acquisition:* two different approaches to acquire contactless FP samples on an iPhone 11 were evaluated. The first one was the default iPhone camera app, and the other one was a customized acquisition solution, the so called T3K app. The evaluation results show that the samples acquired by the T3K app lead to a superior recognition performance, which is mainly because the default iPhone camera app used the built-in auto-focus, which is not capable of accurately focusing on the fingertips, especially if an inhomogeneous background is present (e.g., in outdoor data acquisition scenarios). Another benefit of the T3K app is that the built-in camera light can be turned on permanently, which enables a more uniform illumination of the fingertip area. Hence, the T3K app is the recommended tool for acquiring the contactless FP samples.
- *FP Segmentation:* Several traditional and more recent DL-based algorithms were evaluated. The experiments showed that Mask R-CNN and Deeplab trained on RGB images provided the most accurate segmentation masks, even compared with more recent deep-learning based competitors. If we are interested in bounding boxes rather than masks, SSD MobileNet (Quantized) and Mask R-CNN provide the best results. Due to portability issues of Mask R-CNN, this model could not be evaluated on the iPhone 11. Both Deeplab and SSD MobileNet satisfy the necessary requirements for real-time fingertip annotation on an iPhone 11. However, due to robustness problems when using Deeplab, we recommend the use of SSD MobileNet in the final solution.
- *FP Quality Assessment:* the method used is based on a Canny edge detector, which measures the sharpness of the segmented FP images. If the image is sharp enough, it is further preprocessed. We conducted no further, more detailed analyses because, for our purposes, the applied method turned out to be appropriate and reliable enough. However, we recommend using dedicated FP quality measures in the final solution as they are more capable of measuring the quality of FP specific characteristics. For this purpose, we plan to port the NFIQ2 to the iPhone 11 as well.
- *FP Preprocessing:* two different methods were applied in this work to enhance the FP images that were originally captured: bilateral filtering + CLAHE and average filtering + CLAHE + image sharpening. Furthermore, the resulting greyscale images were either inverted or were not in order to compensate for different light conditions. The second enhancement strategy outperformed the first one, especially when the grey values were inverted. Nevertheless, it is likely that variations introduced during the acquisition, e.g., due to different environmental conditions, will have a severe impact on the observed recognition performance. This issue needs further investigation, and thus, no clear recommendation on the FP preprocessing method can be given.
- *Contactless to contact-based mapping:* this aspect was outside the scope of the current work and we only performed a manual DPI adaptation for the whole dataset rather than on a per-sample basis, and this certainly needs further improvement as no dedicated contactless to contact-based mapping is performed. The recommendation is that a DPI adaptation is definitely necessary, preferably on a per sample basis and in combination with further contactless to contact-based mapping. Both will be evaluated in our future work.
- *Feature Extraction and Comparison*: as mentioned in the Introduction section, one of the requirements is that the solution developed can be integrated into the existing AFIS of the Austrian police. Hence, we can neither change any parameters of the existing feature extraction and comparison step nor introduce any additional modules into their system. The handshake part is where the modified/mapped sample is transferred from our iPhone solution to the police AFIS system and processed there, and a result in the form of a successful identification (or no identification possible) is received. Hence, it does not make sense to give a recommendation for this stage.
- *Final Decision:* as mentioned above, the identification result is received from the police AFIS after it is double-checked by a certified human expert and then displayed to the police officer in the T3K app. At the current stage of the project, this stage of the toolchain has not yet been implemented, and it will be one part of our future work.

To sum up, for a final integrated prototype, we recommend using the proposed T3K app for FP sample acquisition using SSD MobileNet as the FP segmentation method and average filtering + CLAHE + image sharpening to enhance the acquired samples. It is recommended to make improvements to the quality assessment and FP contactless to contact-based mapping stages.

## 5. Conclusions and Future Work

In this paper, we presented an engineering study featuring an in-depth and comprehensive analysis of the toolchain stages necessary for a mobile fingerprint recognition solution, namely image capturing, fingerprint pre-processing, fingerprint contactless to contact-based mapping of fingerprints, and feature extraction and comparison. The goal of this study was to give recommendations for each stage and to provide guidance for further progress towards a final, fully functional prototype of a mobile FP capturing tool for police use in the field, implemented on an Apple iPhone 11, as a part of the BioCapture project, which aims to optimize the existing identification process carried out by Austrian police officers. The most important requirement for this mobile fingerprint solution was the integration into the current police workflow and AFIS system.

To evaluate the different stages of the recognition toolchain, two datasets acquired in a contactless manner in real life scenarios were created. The main focus of our evaluation was on the fingertip detection and segmentation. In addition, we shed some light on different aspects of interoperability: (i) the estimation and correction of the image resolution in terms of DPI, (ii) a first evaluation of the performance of the comparison of contactless FPs with contact-based ones using state-of-the-art FP recognition software, and (iii) the influence of different fingerprint enhancement methods employed during the fingerprint preprocessing stage. To enable this evaluation, contact-based samples from the same subjects were acquired.

It turned out that, for practical applications (outdoor use with an inhomogeneous background), only the DL-based segmentation approaches were suitable. Furthermore, it became apparent that an accurate DPI correction is an essential step towards contactless to contact-based interoperability. Our recognition performance results were promising, but with the current prototype, they were not accurate and reliable enough for operational police use.

### Future Work

The current prototype capturing tool provides the following functionality: FP sample acquisition, fingertip segmentation, image quality estimation, and a simple FP enhancement. Based on the knowledge and insights gained during the engineering study that was performed, several new approaches towards improving the performance of the current prototype to an acceptable level were identified and are scheduled for future work:

- Adaptive illumination enhancement: in our current approach, we utilize the built-in flashlight of the iPhone as the main illumination source to stabilize the ambient illumination conditions (independent of indoor/outdoor and day/nighttime). Thus, the brightness distribution (bright and dark areas on the fingertip) as well as the shape and position of the shadows (ridges and valleys get inverted) depend on the relative position of the finger and the built-in flashlight. As we have seen during our evaluation, this is an issue that needs to be addressed. One possible solution is a color space transformation in order to compensate for the overilluminated and underilluminated areas. Another possibility is to employ DL-based image enhancement methods that can be tailored to our specific use case.
- Improved FP quality assessment: the current quality measure is essentially a contrast/edge-based measure only, and thus, it fails to reliably assess the FP quality in some of the required acquisition settings. The next version of the capturing tool will include a FP-specific quality measure, ideally optimized for contactless FPs (NFIQ and other measures are not suited to contactless FP samples without modification [82]).
- Stitching of high-quality FP areas: currently, each individual fingertip is assessed and processed as a whole. Due to the 3D structure of each finger (cylindrical shape), the peripheral areas are prone to be out of focus and thus often blurred if the central area is in focus and vice versa. To produce a high-quality FP sample, our idea is to split the FP area into five segments, to evaluate the quality of each segment, to continue the acquisition until there is at least one frame of sufficient quality for each segment, and to finally generate the output FP image by stitching those segments with the highest quality.
- Rotation and tilt compensation: in contactless acquisition, all kinds of finger misplacements are likely to occur, with rotation and tilts being the most important ones. These misplacements are hardly present in contact-based FP samples. Hence, to improve the interoperability, it is vital to compensate for the rotations and tilts in contactless samples. We plan to evaluate several approaches that have been proposed in the literature [20,23,24].
- Improved DPI estimation and correction: As the experimental results confirmed, an accurate DPI correction significantly improves the recognition performance for contactless samples. Our current DPI correction is only able to perform a coarse DPI adaptation (several different DPI resolution steps are tested). We plan to implement an improved version that performs an actual DPI estimation based on the ridge and valley structures of the fingerprints [40] and, thus, is sensitive to small changes in the DPI. The DPI information obtained would then be used to adapt the FP sample resolution to 500 DPI on a per-sample basis rather than a per-dataset one.
- Dedicated contactless to contact-based mapping: so far, no dedicated mapping has been implemented. Several studies have shown that, by applying a specific contactless to contact-based mapping, the interoperability can be improved to a great extent [21]. This mapping is also related to rotation and tilt compensation. Many of the proposed methods include the correction step within the mapping [20,23,24]. We plan to implement, adapt, and evaluate several of these methods.
- Runtime performance improvements: While both the DeepLab and SSD-MobileNet models run in real time on the iPhone, a more detailed analysis of the differences in computation time could further improve the performance.

## Figures and Tables

**Table 1:** Main characteristics of the scenarios.

ID-Name	Indoor/Outdoor	Description
1-Office A	Indoor	Artificial light. Homogeneous texture (carpet) in the background.
2-Office B	Indoor	Artificial light. Inhomogeneous texture (table, keyboard, and papers) in the background.
3-Street	Outdoor	Sunny day, in shade. Homogeneous texture (street) in the background.
4-Grass	Outdoor	Sunny day, in shade. Inhomogeneous texture (grass field) in the background.
5-Wall	Outdoor	Cloudy day. Homogeneous texture (wall) in the background.
6-Lattice	Outdoor	Cloudy day. Inhomogeneous texture (lattice) in the background.
7-Cellar	Indoor	Dark room, camera flash on. Homogeneous texture (cement) in the background.

**Table 2:** Average object detection/segmentation performance of the approaches presented in Section 2.2. The scores presented (mean of intersection over unions (mIoUs)) are measured on the test set. Mean/Std are calculated from five training runs.

	Method	Bounding Box		Mask	
		*RGB*	*Greyscale*	*RGB*	*Greyscale*
*Traditional* *Approaches*	Fast and accurate skin segmentation	0.29		0.39	
	Color histogram segmentation	0.27	-	0.38	-
	Gaussian Mixture Model	0.19		0.31	
*Detection* *Approaches*	SSD MobileNet	0.87 (0.03)	0.89 (0.03)	-	
	SSD MobileNet Quantized	0.92 (0.01)	0.92 (0.01)		
*Segmentation* *Approaches*	Mask R-CNN	0.95 (0.00)	0.948 (0.01)	0.96 (0.00)	0.956 (0.00)
	Deeplab	0.88 (0.10)	0.81 (0.14)	0.94 (0.03)	0.93 (0.03)
	Segnet	0.86 (0.03)	0.84 (0.02)	0.90 (0.03)	0.90 (0.01)
	HRNet	0.81 (0.03)	0.60 (0.10)	0.92 (0.01)	0.85 (0.03)

**Table 3:** Dots per inch (DPI)-based Equal Error Rate (EER) results (in %) for both datasets that were used, the enhancement methods (including the optional grey value inversion), and their corresponding DPI settings selected for the VeriFinger SDK (the best results are highlighted in **bold** numbers).

	DPI	AIT Dataset		PLUS Dataset				AIT Dataset		PLUS Dataset			
		*bilateral filtering + CLAHE*						*avg. filtering + CLAHE + img. sharp.*					
		DS84c		G1		V311		DS84c		G1		V311	
		inv.	not inv.	inv.	not inv.	inv.	not inv.	inv.	not inv.	inv.	not inv.	inv.	not inv.
iPhone app	400	-		**6.49**	7.21	8.99	8.90	-		**5.87**	6.04	8.17	8.01
	450			8.84	8.65	**8.57**	8.98			6.78	6.56	**6.62**	7.25
	500			21.47	22.96	17.08	17.52			19.93	21.03	16.84	17.27
T3K app	750	4.46	4.53	15.53	16.73	21.01	20.68	4.41	4.63	16.22	16.76	21.18	21.14
	850	**1.90**	2.00	4.76	5.17	6.03	6.63	**1.65**	1.71	5.18	4.50	5.91	5.92
	950	4.19	4.32	**4.60**	5.40	**5.32**	5.73	3.79	4.22	**4.21**	4.76	**4.71**	5.22

**Table 4:** EER results (in %) for different evaluation settings, which include left/right hand, left/right index + middle finger, and all fingers. This evaluation has only been done for the best DPI setting on the T3K app.

Setting	AIT Dataset	PLUS Dataset		AIT Dataset	PLUS Dataset	
	*bilateral filtering + CLAHE*			*avg. filtering + CLAHE + img. sharp.*		
	DS84c	G1	V311	DS84c	G1	V311
left hand	1.93	4.89	5.71	1.63	4.59	5.01
right hand	2.14	4.90	5.39	1.78	4.26	5.23
left index + middle	1.39	3.51	3.18	1.35	2.93	2.47
right index + middle	0.04	0.91	1.29	0.04	0.48	0.91
left index	0.00	3.53	0.67	0.00	3.53	0.67
left middle	3.63	4.60	4.76	3.47	4.60	5.83
left ring	0.24	8.81	5.56	0.16	8.81	9.76
left pinky	3.80	5.16	10.12	3.30	4.88	5.27
right index	0.16	0.00	2.58	0.00	0.00	0.67
right middle	0.00	2.05	0.97	0.00	0.97	1.26
right ring	0.00	11.67	10.43	0.00	9.60	10.56
right pinky	7.19	8.28	10.34	6.03	8.16	8.56

**Table 5:** Computation time analysis for three different scenarios. The reported numbers denote the time required for annotating a single video frame.

Scenario Nr.	Statistic	Deeplab [ms]	SSD MobileNetV2 [ms]
All	Minimum	34.78	20.87
	Maximum	44.94	42.56
	Median	38.65	24.97
	Mean	38.73	25.41
	Std. Dev.	1.52	2.67
1	Minimum	34.70	20.66
(Indoor)	Maximum	44.46	42.39
	Median	37.88	23.74
	Mean	38.13	24.35
	Std. Dev.	1.49	2.35
4	Minimum	34.82	20.87
(Outdoor)	Maximum	44.30	44.80
	Median	38.06	24.67
	Mean	38.25	25.50
	Std. Dev.	1.36	3.09

**Figure A4:**
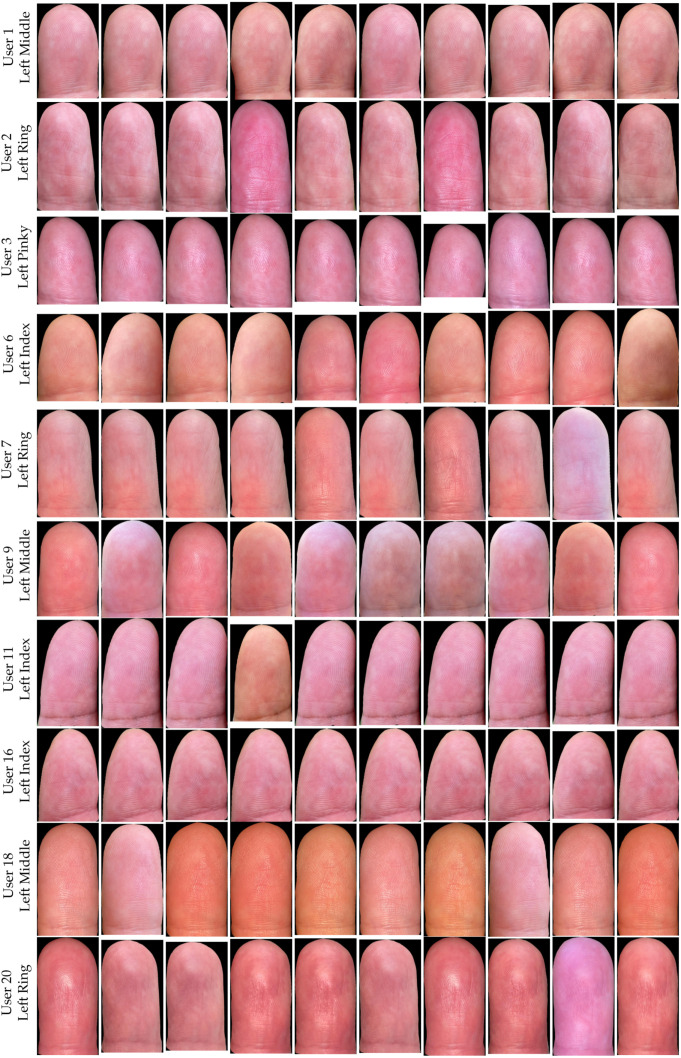
Fingertip samples obtained from the AIT dataset.

## Abbreviations

The following abbreviations are used in this manuscript:

ADAM	Adaptive Moment Estimation
AFIS	Automated Fingerprint Identification System
AIT	Austrian Institute of Technology
API	Application Programming Interface
CNN	Convolutional Neural Network
COTS	Commercial Off The Shelf
DL	Deep Learning
DPI	Dots Per Inch
EER	Equal Error Rate
FAR	False Acceptance Rate
FBI	Federal Bureau of Investigations
FMR	False Match Rate
FNMR	False Non-Match Rate
FP	Fingerprint
FPN	Feature Pyramid Network
FRR	False Rejection Rate
FVC	Fingerprint Verification Contest
GDPR	General Data Protection Regulation
GPU	Graphics Processing Unit
HSV	Hue Saturation Value
MDPI	Multidisciplinary Digital Publishing Institute
ML	Machine Learning
NFIQ2	NIST Fingerprint Image Quality Version 2
NIST	National Institute of Standards and Technology
PLUS	Paris Lodron University Salzburg
RGB	Red Green Blue
SDK	Software Development Kit
SGD	Stochastic gradient descent
SSD	Single Shot Detector
ST	Saturation and Lightness
TSL	Tint, Saturation and Lightness
